# Complete spatiotemporal quantification of cardiac motion in mice through multi-view magnetic resonance imaging and super-resolution reconstruction

**DOI:** 10.1038/s41598-025-11766-5

**Published:** 2025-08-13

**Authors:** Tanmay Mukherjee, Maziyar Keshavarzian, Elizabeth M. Fugate, Vahid Naeini, Amr Darwish, Jacques Ohayon, Kyle J. Myers, Dipan J. Shah, Diana Lindquist, Sakthivel Sadayappan, Roderic I. Pettigrew, Reza Avazmohammadi

**Affiliations:** 1 Department of Biomedical Engineering, Texas A&M University, College Station, TX 77843 USA; 2 Department of Radiology, Cincinnati Children’s Hospital Medical Center, Cincinnati, OH 45229 USA; 3 Houston Methodist DeBakey Heart & Vascular Center, Houston, TX 77030 USA; 4 Savoie Mont-Blanc University, Polytech Annecy-Chambéry, Le Bourget du Lac, France; 5 Laboratory TIMC-CNRS, UMR 5525, Grenoble-Alpes University, Grenoble, France; 6 Hagler Institute for Advanced Study, Texas A&M University, College Station, TX 77843 USA; 7 Department of Internal Medicine, Division of Cardiovascular Health and Disease, University of Cincinnati College of Medicine, Cincinnati, OH 45267 USA; 8 School of Engineering Medicine, Texas A&M University, Houston, TX 77030 USA; 9 Department of Cardiovascular Sciences, Houston Methodist Academic Institute, Houston, TX 77030 USA; 10 J. Mike Walker ’66 Department of Mechanical Engineering, Texas A&M University, College Station, TX 77843 USA

**Keywords:** Small animal cardiac magnetic resonance, Super-resolution reconstruction, In-silico phantom, Strain estimation, Diagnostic and prognostic tool, Biomedical engineering, Diagnostic markers

## Abstract

Background: Structural indices of cardiac diseases estimated via cardiac magnetic resonance imaging (CMR) have shown promise as early-stage markers. Despite the growing popularity of CMR-based myocardial strain calculations, measures of complete spatiotemporal strains (i.e., three-dimensional strains over the cardiac cycle) remain elusive, especially in mice. The high metabolic rates and rapid cardiac motion affect high-resolution imaging, thus compromising strain accuracy. We hypothesize that a super-resolution reconstruction (SRR) framework that combines low-resolution scans at multiple orientations will enhance the reliability of complete spatiotemporal strains in mice. Methods: Multi-view cine CMR comprising short- and long-axis (SA and LA) fast low angle shot scans were obtained in a cohort of wild-type-mice (n = 5) and a diabetic mouse (n = 1). The “SRR in CMR” approach, consisting of tissue-class -specific scattered data interpolation, was used to generate full four-dimensional (4D) images of high spatial resolution. Image registration using the diffeomorphic demons algorithm was applied to quantify complete spatiotemporal motion in terms of 4D myocardial strains. The effects of SRR on CMR quality were verified in all mice through image metrics, namely, root mean squared error (MSE) and structural similarity index. Strain calculations were validated against an in silico heart model phantom through MSE analysis, followed by investigations of strain accuracy and reproducibility for all mice using MSE and coefficient of variation analyses. Results: SRR-derived strains were validated against a kinematic benchmark established through the in-silico heart model phantom. Improvements in global strain accuracy were confirmed in both in-plane (radial and circumferential) and through-plane (longitudinal) strains. Mouse-specific SRR provided near isotropic spatial resolution, high structural similarity, and minimal loss of contrast, which led to overall improvements in strain reproducibility and intra-cohort homogeneity in wild-type mice, with global longitudinal strain lying of ≈-14%. Conclusions: A comprehensive methodology was presented to quantify complete and reproducible myocardial deformation, aiding in the much-needed standardization of complete spatiotemporal strain analysis in small animals.

## Introduction

Functional assessments of the left ventricle (LV) have become crucial in the diagnosis of structural heart diseases^1^. Organ-level measurements, such as LV ejection fraction (EF) and end-diastolic volume, are key determinants of systolic and diastolic dysfunction^1,2^. However, the diagnostic and prognostic efficacy of these “global” indices is challenged by the multi-faceted nature of structural heart diseases. These metrics lag as indicators for most cases of early-stage pathology, with additional investigations often required for risk stratification^3,4^. The analysis and subsequent characterization of myocardial strains are one such investigation used to complement global metrics. The image-based evaluation of myocardial deformation through global longitudinal strains (GLS) has been shown to be a stronger determinant of cardiac diseases at an early stage in comparison with traditional volumetric metrics^5–8^. Despite their merits, such organ-level strain measurements fail to quantify the regional characteristics of LV function accurately. The LV exhibits complex four-dimensional (4D) kinematics, i.e., full three-dimensional (3D) deformation over the cardiac cycle with remarkable transmural variations, thus confounding the accuracy of regional strain calculations and effectuating variability^9,10^. The characterization of continuous 4D regional deformation offers excellent potential in establishing a complete LV structure-function relationship. Despite advances in medical imaging, the assessment of 4D continuous regional myocardial deformation remains under-explored.

The exceptional soft tissue contrast offered by cardiac magnetic resonance imaging (CMR) has presented itself as a vital resource in the estimation of regional strains. Tissue tagging methods using specialized image sequences have become the most validated strain calculation strategy. However, the complex protocols involved in tag deposition and tracking may pose challenges, especially when 4D strain calculations are of interest^11^. Alternatively, studies on strain calculation using motion-tracking approaches such as image registration, feature-tracking, and optical flow have been implemented wherein cardiac motion is computed using standard cine CMR images of the LV short- and long-axis (SA and LA, respectively). Interpolation schemes can be used to derive spatially 3D strains by extrapolating strains estimated at the epi- and endocardium^12^. However, standard imaging protocol involving a single LA scanning plane may not be sufficient to estimate regional 3D strains resulting from significant through-plane motion due to the presence of combined torsional and longitudinal motion in the heart^13^. Indeed, although we have previously shown that stacks of short-axis (SA) scans can be potentially used to estimate 4D myocardial strains using image registration^14,15^, a spatially dense stack of SA scans is expected to be needed to describe through-plane motion. An alternative to acquiring dense stacks of SA scans is the implementation of super-resolution reconstruction (SRR) to generate high-resolution (HR) images from a stream of low-resolution (LR) images. The ability to choose scanning planes at various orientations enables the feasibility of SRR in CMR, and applications are growing in prevalence^16–20^. Odille et al.^17^ suggested a slice-to-volume reconstruction approach which involved an ad-hoc modification of LR images during acquisition, and Rahman et al.^16^ used combined acquisitions of SA and LA views of the LV in their reconstruction methodology. However, finite deformation due to the rapid motion of the cardiac wall remains a major challenge in implementing SRR for small animals.

Indeed, the contraction of the heart results in remarkable heterogeneous motion, determined by factors such as heart rate, preload, and afterload, amongst others. Deep-learning (DL) -based SRR using arbitrarily oriented LR images have been suggested in combating frame registration errors due to high-speed cardiac motion^21,22^. Investigations have revealed significant improvements in image quality and diagnostic reliability. The improvement of image reconstruction and subsequent reduction in anisotropy^21–23^ offers significant potential in the complete quantification of 4D myocardial motion. Xia et al.^24^ remarked upon the potential benefits of SRR in myocardial strain analysis and suggested that a combination of poor spatiotemporal resolution and signal-to-noise ratio (SNR) must be addressed to reduce variability in strain calculations. Therefore, there is a need for advancing SRR techniques to improve the reproducibility of the complete spatiotemporal myocardial strains, herein referred to as 4D strains. An impediment in DL-based SRR frameworks is the requirement of ground-truth HR images and motion^25^. Importantly, the heart exhibits strong subject-specific variations in contractile patterns in healthy and diseased states, thus also demanding extensive data repositories. Finally, despite recent efforts in implementing unsupervised learning methods to create SR images training the ML model in humans^24,26^, applications of SRR in small animals remain limited.

**Fig. 1:**
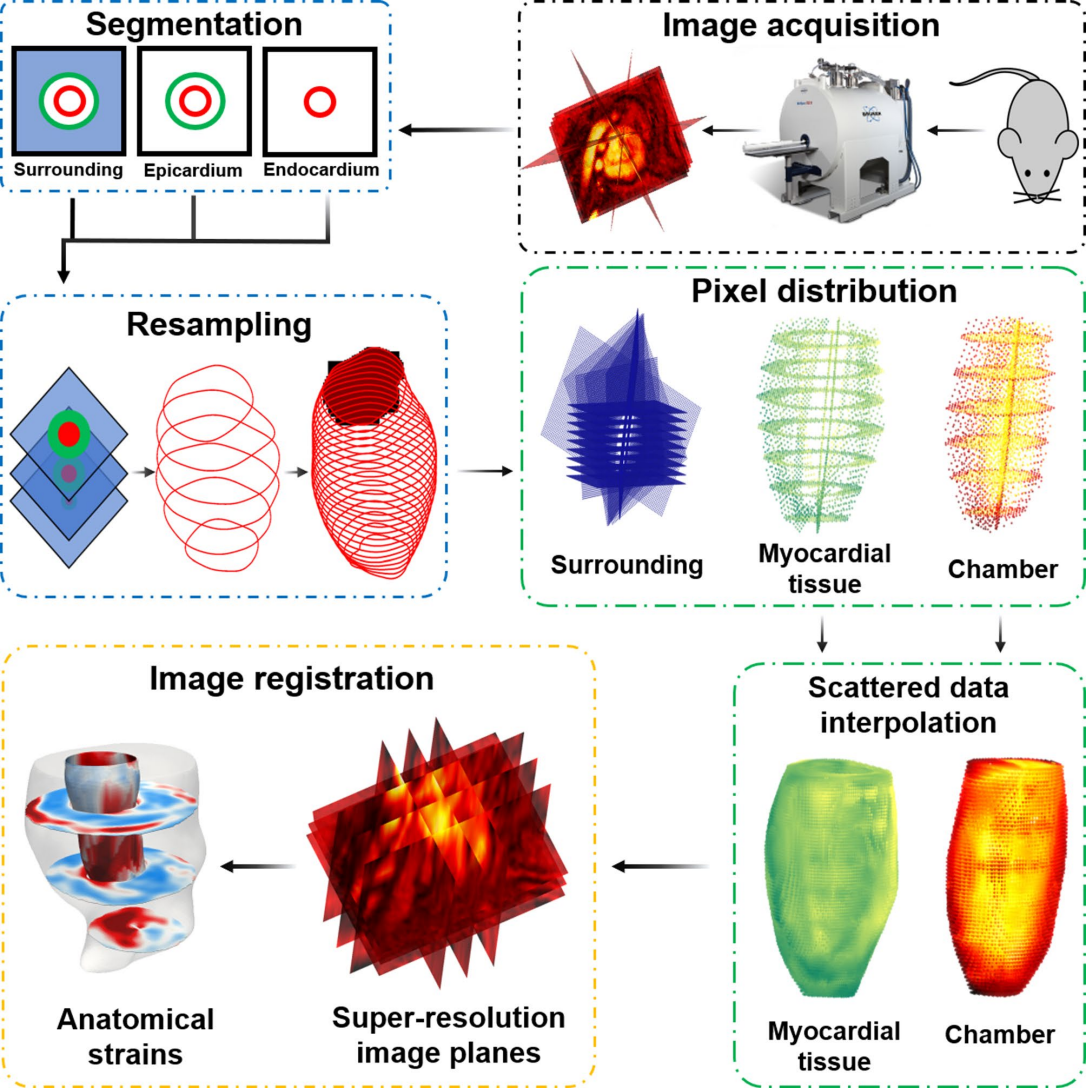
The architecture of the super-resolution reconstruction (SRR) in the cardiac magnetic resonance (CMR) framework consists of (1) image acquisition, (2) SRR, (3) non-rigid image registration, and (4) anatomical strain calculations. Blue line: classification module, Green line: interpolation module, and Brown line: registration module.

In this work, we propose a methodology to produce and validate 4D strains in mice using cine CMR imaging and SRR through scattered data interpolation. Our method updates the classical SRR problem by classifying pixels in each LR scan based on tissue type to improve the spatial resolution within distinct regions of the heart. We hypothesize that the reproducibility of complete spatiotemporal strain calculations can be promoted through the improvement of the distinction between each region, i.e., tissue class, and improved spatial resolution. First, an in-silico study was conducted using a heart model phantom to guide the placement of SA and LA scanning planes to represent the strongly heterogeneous 4D strains. The phantom study was specifically used to optimize the cine CMR protocol with respect to (i) geometric reconstruction, (ii) strain accuracy, and (iii) strain reproducibility. Following this, the method was applied to mouse-specific images, as the high heart rates present a stringent testbed for assessing strain fidelity and temporal resolution. Strains were derived from synthetic and mouse-specific images using the proposed “SRR in CMR” approach, which combines sets of arbitrarily aligned anisotropic LR scans to produce a user-defined 3D HR scan (Fig. 1). Following SRR, a non-rigid image registration algorithm was used to calculate pixel displacements and derive 4D strains. Subsequently, strain accuracy and reproducibility were evaluated in a cohort of mice. The SRR-derived strains were also validated using the kinematic benchmark established through the in-silico heart model phantom. Our findings demonstrated the recovery of expected global patterns of LV shortening, a reduction in physiologically implausible strain values, improved intra-cohort homogeneity of myocardial strains, and decreased through-plane anisotropy, with minimal degradation of tissue contrast.

## Methods

### Development of SRR in CMR

#### Cardiac tissue classification

The reconstruction of an HR image from sets of LR images can be formulated as an inverse problem. The LR images can be taken as a set of observations($\textbf{y} \mathrm {=\{}\textbf{y}\mathrm {_{1},}\textbf{y}\mathrm {_{2},...}\textbf{y}\mathrm {_{n}\}}$), and the HR image (${\textbf {x}}$), is the ideal undegraded discrete representation of the real scene. Each representation can be formulated by combining $\textbf{x}$ and random noise, denoted by $\bf{\eta}$. Specifically, linear additive Gaussian noise can be assumed when the SNR is greater than three for CMR images^14,27^. Subsequently, the vectorial representation can be formulated as:

1 $$\begin{aligned} {\textbf {y}}_k = {\textbf {A}}_k { {\textbf {x}}_k} + \bf{\eta }_k \hspace{0.1in}, \end{aligned}$$

where $\textbf{A}_k$ describes the image acquisition process for the $k^{\text {th}}$ observation. Each observation is a subsampled version of $\textbf{x}$, which is subject to various imaging conditions – namely downsampling, geometric warping, and blurring ( $\textbf{D}\text{ ,}\textbf{W}$ , and $\textbf{B}$ respectively). We can decompose the acquisition model as $\textbf{A}_k = \textbf{D}_k \textbf{B}_k \textbf{W}_k$ ^16,28^. Thus, for a given set of observations (${\textbf {y}}$), an optimization problem is posed to calculate the undegraded HR image (${\textbf {x}}$) as:

2 $$\begin{aligned} \textbf{x} = \underset{\textbf{x}}{\text{argmin}} \sum _{k=1}^{n} \left\| \textbf{y}_{k} - \textbf{A}_{k} \textbf{x}_{k} \right\| ^2 \hspace{0.1in}. \end{aligned}$$

This classical SRR problem can be divided into three steps: (i) registration of the LR images, (ii) interpolation onto a structured or unstructured HR grid, and (iii) deconvolution to remove noise and blur. However, the implementation of this model for CMR images is challenged by the cyclical movement of the cardiac muscle and sharp differences in contrast between the blood pool and the myocardial tissue. To address this limitation, each LR observation was divided into three distinct regions, and an optimization problem was posed to generate the undegraded HR observation of a single tissue class (*j*) from a series of LR images. Herein, the regions were classified based on mean gray level intensity into (i) the blood pool (chamber), (ii) myocardial tissue, and (iii) the surroundings. Our objective was to categorize each *j*, and interpolate from the set of the LR images onto a predefined HR grid consisting of numerous equally spaced query points on a global Euclidean space as:

3 $$\begin{aligned} {\textbf {x}} = \underset{\textbf{x}}{\text {argmin}} \Bigg \{ \sum _{j=1}^3 \sum _{k=1}^{n} \Vert {\textbf {y}}_{j}^{k} - {\textbf {A}}_{j}^{k} {\textbf {x}}_{j}^{k} \Vert ^2 \Bigg \}\hspace{0.1in}. \end{aligned}$$

Pixel locations corresponding to each *j* were isolated via a combination of (i) contours drawn on 2D images and (ii) connectivity information describing the region in a structured 3D space, i.e., the Cartesian coordinates. Contours were drawn semi-automatically^29^ to segment the endocardium and epicardium using Segment version 3.0^30^. First, pixels corresponding to each *j* were located through an image histogram to guide contouring. These 2D contours were resampled longitudinally through Delaunay triangulation. In essence, each location was treated as a point and connected through triangulation, resulting in a 3D iso-surface. Subsequently, radial basic functions were applied to smooth the segmentation in order to limit the effects of spatial anisotropy on overall segmentation.

#### Building tissue class-specific interpolants

A structured HR grid was initialized and the approximate locations of each *j* was determined using the prior information of the mean gray-level intensity. The objective was to promote smooth transitions across *j* and guide the reconstruction toward more plausible image gradients based on known tissue characteristics. Specifically, this prior information was incorporated into the SRR framework through a regularization term added to the right-hand side of the modified SR equation (Eq. 2) as:

4 $$\begin{aligned} {\textbf {x}} = \underset{\textbf{x}}{\text {argmin}} \Bigg \{ \sum _{j=1}^3 \sum _{k=1}^{n} \Vert {\textbf {y}}_{j}^{k} - {\textbf {A}}_{j}^{k} {\textbf {x}}_{j}^{k} \Vert ^2&+ \lambda \sum _{j} \sum _{k=1}^{n} \left( \frac{{\textbf {x}}_{j}^{k} - \mu _{j}}{\sigma _{j}} \right) ^2 \Bigg \}\hspace{0.1in}, \end{aligned}$$

where $\mu _{j}$ and $\sigma _{j}$ represent the mean and standard deviation gray level intensity of each *j*, respectively, and $\lambda$ represents the regularization parameter. Thus, deviations from the mean gray level intensity are penalized, which is imperative in improving image registration. The optimization problem (Equ. 4) was used to form region-specific or tissue-class-specific interpolants to solve for ${\textbf {A}}_k$. The segmented LR images, along with the information describing the desired HR scan, were passed on to the interpolation module. Since images are projected onto either the SA or the LA planes, the coordinates describing the pixel intensities of each image were obtained by accessing the position and direction cosines from the DICOM metadata. The target vector describing $k^{\text{th}}$ observation of an LR image was thus obtained, and the rotation matrix was derived using Rodrigues’ rotation formula as:

5 $$\begin{aligned} {\textbf {R}}_{ k} = {\textbf {I}} + {\textbf {S}}_{ k} \text{sin} \phi + (1 - \text{cos} \phi ) {\textbf {S}}^{ 2}_{ k} \hspace{0.1in} ; \hspace{0.5in} {\textbf {S}}_{ k} {\textbf {v}}_{ k} = {\textbf {r}}_{ k} \times {\textbf {v}}_{ k}, \end{aligned}$$

where ${\textbf {R}}$ is the rotation matrix that uses the skew-symmetric transformation matrix *S* obtained from the cross product of the vector ${\textbf {v}}$ and the axis of rotation ${\textbf {r}}$ to rotate the image by an angle $\phi$, and $\textbf{I}$ is the identity matrix. The rotated image was translated using the position metadata, and the coordinate space describing all observations of the LR images was obtained. Subsequently, the HR scan was allowed to be defined as a structured grid via a global interpolation scheme. Given the anisotropy in pixel distribution and to accommodate arbitrary orientations of LR images, scattered data interpolation was used to estimate ${\textbf {A}}_k$. The global interpolant consisted of individual regional coordinates interpolated based on their tissue classes. The module allows two regional and global interpolation schemes, namely, (a) *linear*, wherein linear interpolation is used for reconstruction, and (b) *natural* or *pchip*, wherein the HR grid for each tissue class is defined using Delaunay triangulation-inspired piecewise cubic Hermitian polynomials. Each projection of the LR image was evaluated systematically to reduce blurring, and gaps in space were filled over the pre-defined HR grid using the global interpolation scheme, thus yielding a stack of SR images.

#### Calculating time-course cartesian displacements

The LR and HR grids, corresponding intensity maps, and the combined package comprising parameters such as the resampling ratios, the reference image index, and the segmented contours were used to perform motion calculations using image registration. A diffeomorphic demons algorithm was implemented to perform non-rigid image registration^14^. The algorithm aligns a moving image ${\mathscr {M}}$ to the fixed image ${\mathscr {F}}$ through the minimization of the global energy function, $\mathcal{W}$ as:

6 $$\begin{aligned} {\mathcal{W}}({\textbf {c}}, {\textbf {u}}) = \frac{1}{\sigma _{{\textrm{i}}}^{2}}\Vert \mathscr {F} - \mathscr {M} \circ {\textbf {c}}\Vert ^2 +\frac{1}{\sigma _{{\textrm{x}}}^{2}}\Vert {\textbf {u}} - {\textbf {c}}\Vert ^2 + \frac{1}{\sigma _{{\textrm{T}}}^{2}}\Vert \nabla {\textbf {u}}\Vert ^\textrm{2}, \end{aligned}$$

where ${\sigma _\textrm{i}}$, ${\sigma _\textrm{x}}$, and ${\sigma _\textrm{T}}$ are the noise intensity, spatial uncertainty, and regularization factor, respectively, and $\textbf{u}$ and $\textbf{c}$ denote the parametric and non-parametric spatial transformations, respectively. The registration was performed over three pyramid levels with 500, 400, and 300 iterations.

#### Estimating anatomical strains

The image registration-derived pixel displacements in the Cartesian frame of reference were used to calculate the Green-Lagrange strain tensor, $\textbf{E}$. Strains were estimated between each reference frame ($\textbf{x}$) and the target frame ($\textbf{X}$) through the deformation gradient tensor, $\textbf{F}$, as:

7 $$\begin{aligned} {\textbf {E}} = \frac{1}{2} \left( {\textbf {F}}^{T} {\textbf {F}} - {\textbf {I}} \right) , \end{aligned}$$

The gradient $\textbf{F}$ was calculated as the propagation of the deformation gradient between two consecutive load increments ($\mathbf {F_{\textrm{i}}}$) such that,

8 $$\begin{aligned} {\textbf {F}} = \prod _{i} {\textbf {F}}_{ i} \hspace{0.1in}; \hspace{0.1in} {\textbf {F}}_{ i} = {\textbf {I}} + \frac{\partial {\textbf {u}}}{ \partial {\textbf {X}}} \hspace{0.1in} = \left[ \begin{array}{lll} \frac{\partial u_{x}}{\partial X} & \frac{\partial u_{x}}{\partial Y} & \frac{\partial u_{x}}{\partial Z} \\ \frac{\partial u_{y}}{\partial X} & \frac{\partial u_{y}}{\partial Y} & \frac{\partial u_{y}}{\partial Z} \\ \frac{\partial u_{z}}{\partial X} & \frac{\partial u_{z}}{\partial Y} & \frac{\partial u_{z}}{\partial Z}\\ \end{array} \right] , \end{aligned}$$

where ${u_{x}}$, ${u_{y}}$, and ${u_{z}}$ are the displacements in the x,y, and z directions between two consecutive images. The resulting Cartesian strains were converted to the widely used anatomical or radial-circumferential-longitudinal (RCZ) axes using an orthonormal transformation matrix ($\textbf{Q}$) as:

9 $$\begin{aligned} {\textbf {E}}_{[R,\Theta ,Z]} = {\textbf {QEQ}}^{T} \hspace{0.1in} = \left[ \begin{array}{lll} E_{RR} & E_{R \Theta } & E_{RZ} \\ E_{\Theta R} & E_{\Theta \Theta } & E_{\Theta Z} \\ E_{ZR} & E_{Z \Theta } & E_{ZZ} \end{array} \right] , \end{aligned}$$

where $E_{RR}$, $E_{\Theta \Theta }$, and $E_{ZZ}$ are the radial, circumferential, and longitudinal strains, respectively. Finally, the calculated strains were mapped onto a reconstructed geometry of the LV obtained through segmentation to facilitate the visualization of 4D strains. This visualization is achieved through Delaunay triangulation of all the pixels and strain quantities pertaining to the LV.

### In silico heart phantom experiments

In silico experiments were conducted using a heart model phantom to validate strains generated through SRR and determine optimal scanning parameters to guide mouse-specific imaging. Synthetic images of the heart were synthesized from 3D finite element (FE) simulations of mouse-specific cardiac contraction using the method described extensively by Mukherjee et al^15^. Briefly, the mouse-specific FE geometry was converted into an unstructured visualization toolkit (VTK) standard grid of tetrahedral elements. Individual VTK grids were obtained for all timepoints in the cardiac cycle using the FE-derived Cartesian displacement vectors. These grids were sectioned at incremental positions along the (i) z-axis (normal: $\textbf{n} = [0,0,1]$) to obtain SA slices and (ii) the corresponding orthogonal axis ($\textbf{n} = [1,1,0]$) to obtain LA slices. The slices were subsequently rasterized onto a uniform grid (i.e., image) of predetermined parameters (spatial and temporal resolution, contrast, and slice thickness). Artificial SNR was added by convolving the grids with a Gaussian kernel ($\sigma = 3$). Subsequently, two configurations of synthetic multi-view cine CMR phantoms were obtained through (i) conventional reconstruction, involving linear interpolation using a stack of SA images and (ii) SRR using a combined stack of SA and LA images. Both phantoms were subjected to NRIR using the diffeomorphic demons algorithm and myocardial strain calculations. An ablation study was conducted using the SRR phantoms to determine the most suitable combination of SA and LA slices required to improve strain accuracy.

### Application of SRR to the murine heart

Two distinct subject-specific imaging protocols were devised to estimate 4D cardiac motion in male C57BL/6 mice. The objective of the first protocol was to evaluate the effectiveness of the proposed SRR framework in improving myocardial strain reproducibility, while the second aimed to evaluate its effectiveness in reducing strain variability across a homogeneous cohort. For both studies, mice were anesthetized with inhaled isoflurane with pure oxygen carrier at light sedation (0.5-1%) to maintain near physiological heart rates of 400-500 beats per minute. The animals were euthanized under isoflurane anesthesia by exsanguination post-imaging. The experimental protocols were approved by the Cincinnati Children’s Hospital Medical Center Animal Care and Use Committee (Protocol 2018-0054). Animal use (including anesthesia and imaging) was subsequently conducted in accordance with institutional guidelines. The study is reported in accordance with the ARRIVE guidelines.

#### Image acquisition

**Fig. 2:**
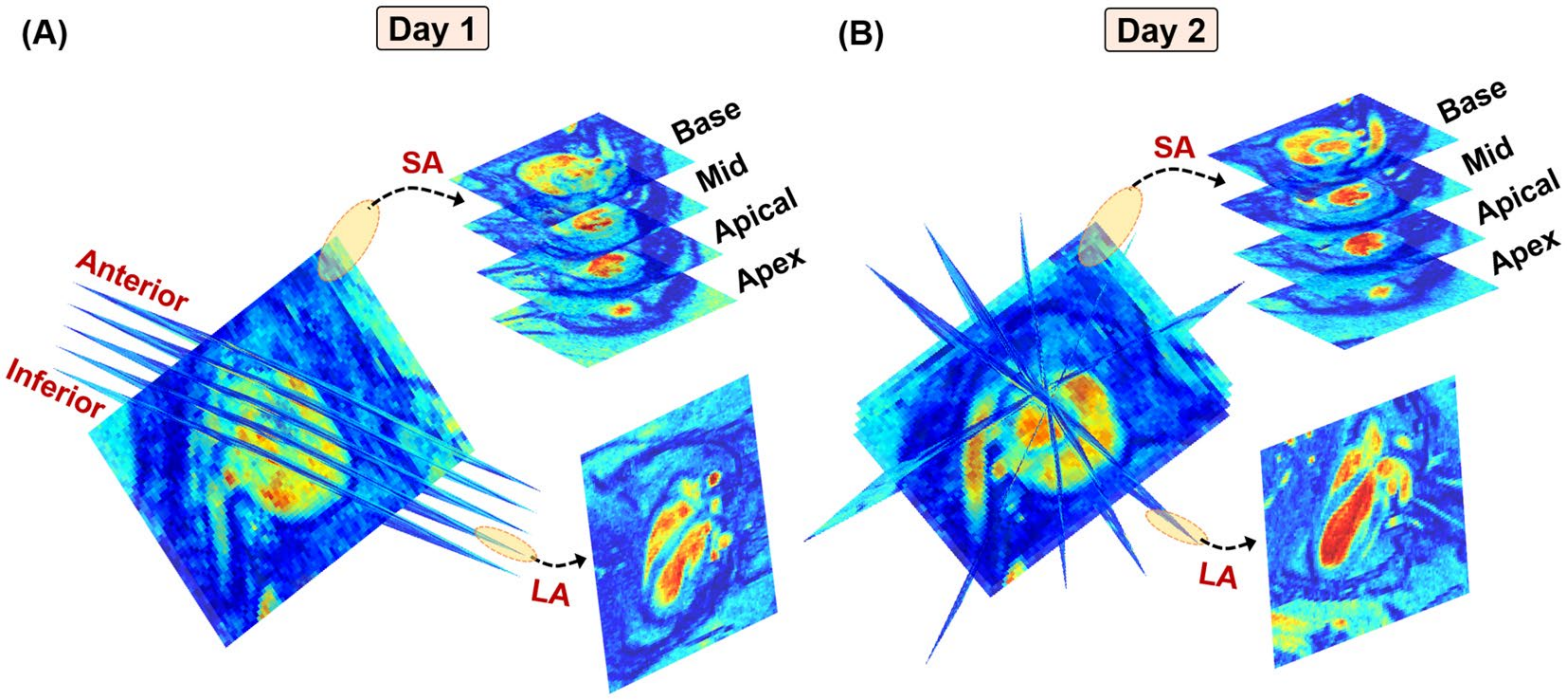
Imaging protocols used in the acquisition of cine CMR images for a three-month-old diabetic mouse over a period of two consecutive days. Combination of eight short-axis (SA) image planes between the basal slice and the apex of the left ventricle (LV) combined with five long-axis (LA) slices sampled (**A**) orthogonally between the anterior and inferior walls of the LV on the first day and (**B**) radially about the LV chamber on the next day.

***Protocol 1:*** Imaging was performed in a three-month-old healthy wild-type (WT) mouse (n = 1) and a three-month-old diabetic mouse (n = 1)^31^. Two different combinations of SA and LA scans were designed, with each set acquired one day apart. On the first day, five LA scans were acquired orthogonally, i.e., scans were acquired through translation between the anterior and inferior walls of the LV (Fig. 2A). The following day, five LA scans were acquired by radially sampling different image planes i.e., scans were acquired through rotating about the LV chamber (Fig. 2B). On both days, eight SA scans describing the region between the basal section of the LV and apex were acquired. ***Protocol 2:*** Imaging was performed in a cohort of five six-month-old WT mice (n = 5). These mice were subjected to similar SA imaging, with LA scans acquired solely via orthogonal sampling. In both protocols, cine CMR imaging was performed using a 7 T Bruker Avance III HD scanner with a 72 mm quadrature proton volume coil (Bruker BioSpin MRI GmbH). In both protocols, an electrocardiogram (ECG) signal was used to trigger image acquisition (R-R over three periods, i.e., three cardiac cycles), and the combined SA-LA set was acquired in one session. ECG and respiration were continuously monitored during imaging. The scan parameters were as follows: Fast Low Angle Shot, TE =1.6 ms, TR = 9 ms, flip angle = $20^{\circ }$, readout bandwidth = 65789 Hz, resolution = 200 $\upmu$m, slice thickness = 1 mm, field of view = 32 x 32 mm, averages = 4.

#### Myocardial strain estimation

Through the image acquisition protocols, various combined SA-LA sets were subjected to SRR, and subsequently, pixel-level displacements and strains were calculated at end-systole (ES), with end-diastole (ED) as the reference frame. Myocardial strains were evaluated separately for SR images, herein referred to as the SR model and the conventionally reconstructed LV. Here, conventional reconstruction refers to the use of image stacks that only contain LR SA images of the LV, herein referred to as the LR model. 2D cubic interpolation was used to increase the in-plane resolution of these LR images to match the in-plane resolution of the SR images. Each LR image was subjected to smoothing using a Gaussian kernel ($\sigma = 2$) before image registration. Unlike the SR images, longitudinal query points for the conventionally reconstructed images were determined strictly based on the aspect ratio of the SA scans^14^. Additionally, the SR grids resulting from the SA-orthogonal LA and SA-radial LA image stacks, herein referred to as SR-O and SR-R, respectively, were also used to calculate myocardial strains. In addition to mapping strains on murine-specific LV geometries, strains were also mapped in American Heart Association (AHA) standard segmentation maps, wherein the basal, mid, and apical sections of the LV were divided into 16 unique segments^32^.

### Statistical analysis

The ablation study consisted of mean squared error analysis to determine the accuracy of the image-derived strains against the benchmark in-silico phantom^15^. The objective was to determine the most suitable configuration to provide reproducible strain patterns. Image quality was analyzed using image acutance metrics, as image artifacts on 4D myocardial strain analysis limit strain reproducibility (Appendix A1). Finally, regional and global strains were analyzed for the WT mice (n = 5) using GraphPad Prism 9 to investigate intra-cohort strain homogeneity. Strains derived from both SR and LR images were visualized using AHA segmentation maps. For each of the 16 AHA segments, the mean squared error between SR- and LR-derived estimates of $E_{\Theta \Theta }$ and $E_{ZZ}$ was computed. Spatial heterogeneity across segments was then evaluated using the coefficient of variation (CV). A paired Student’s t-test was applied to assess the statistical significance and confidence intervals of differences in strain measurements. Furthermore, GLS, calculated as the mean of the 4D $E_{ZZ}$ values, was compared between SR and LR images to evaluate global agreement.

## Results

### Reduction in through-plane anisotropy

**Fig. 3:**
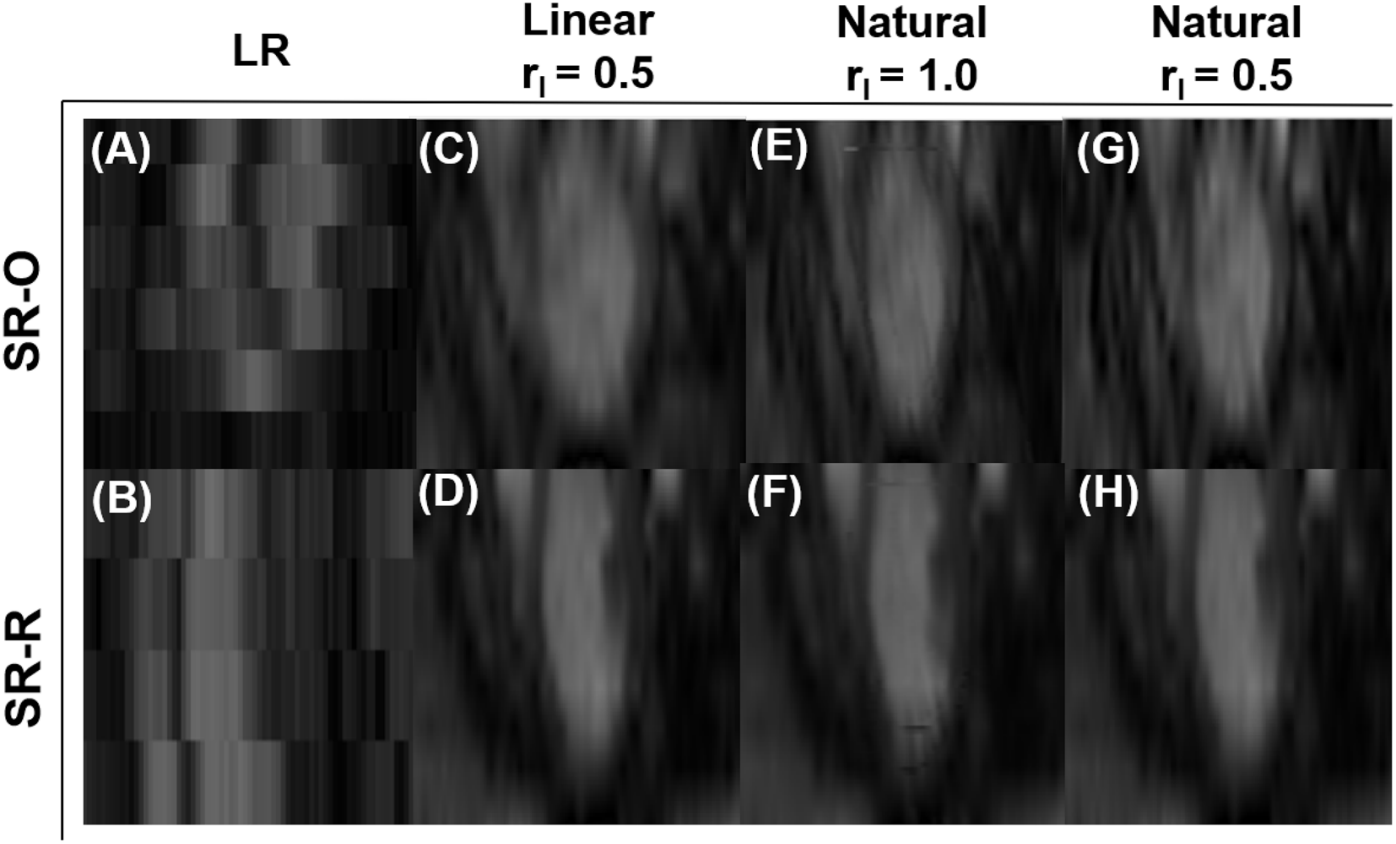
Through-plane reconstruction of an arbitrary plane perpendicular to the LV short-axis (SA), by (**A**, **B**) stacking only the eight low-resolution (LR) SA image planes one over the other, (**C**-**H**) via super-resolution reconstruction (SRR) using (**C**, **D**) *linear* interpolation and (**E**-**H**) *natural* interpolation. SR-O: SRR using the combination of SA and orthogonally sampled LA images; SR-R: SRR using the combination of SA and radially sampled LA images. $\mathrm {r_{1}}$: resampling ratio.

The fundamental consideration of the SRR framework was to increase the spatial resolution of the CMR image stacks, with the benefits of SRR in through-plane LV reconstruction reported in this section. The reconstructed LV is shown across an arbitrary plane sampled perpendicular to the SA planes (Fig. 3). Since only eight LR SA slices were used in the image acquisition process, the LV reconstruction using LR images was hampered considerably by the slice thickness (Figs. 3A, B). Whereas in both cases of SR images, the pixel spacing increased from 140 x 140 x 8 to (i) 140 x 140 x 110, for in-plane resampling ratio $\mathrm {\left( r_{1} \right)} = 1.0$, and (ii) 280 x 280 x 220, for $\mathrm {r_{1}} = 0.5$, respectively. Consequently, any number of planes could be sampled (Supplementary Fig. S1) without being restricted to the orientation of the original LA scanning planes. As expected, there were no discernible differences between the LV chamber and the myocardial tissue along the through-plane direction of the LR images. In contrast, reconstruction was improved using SRR with a clear distinction between the high contrast blood pool and the relatively low contrast myocardial tissue (Figs. 3C-H) throughout the cardiac cycle (Supplementary Fig. S1). However, when the in-plane resolution was limited to $\mathrm {r_{1}} = 1.0$, a few artifacts were encountered in the reconstruction process (Fig. 3E, F). Despite a minor presence of artifacts, both SR protocols outperformed the LR image-derived LV reconstruction, irrespective of the resampling ratio or nature of the global interpolation scheme.

### Improvement in tissue definition

#### Qualitative assessment

For both sets of SR models, the reconstruction of the epicardial and endocardial borders outperformed the LR models. Despite a pronounced improvement in the sharpness of the endocardial and epicardial borders, neither SR model was determined to have produced a perfect separation between the chamber and the myocardial tissue (Supplementary Fig. S2). This was expected as radial basis functions were used at the borders to regularize SNR-related errors. The most well-defined borders were produced through the *natural* interpolation scheme consistently, but this improvement in border definition was accompanied by a marked increase in artifacts, especially in SR-R (Supplementary Figs. S2K, N-P). A few gradient discontinuities in the form of lines were observed over the SRR models. These lines were identified to be repercussions of isometric projections of the LR images onto the HR grid. These discontinuities were restricted to the original positioning of the LA arrangement. However, under the absence of in-plane resampling ($\mathrm {r_{1} = 1.0}$), artifacts were observed over the entire image plane (Supplementary Figs. S2C, K, O). The artifacts are reflected in the respective Likert scores, with the LR images outperforming SRR in this aspect as expected (Appendix; Table A1). Despite these artifacts, both SR models yielded overall improvements in both in-plane and through-plane reconstructions of the LV. However, qualitative comparisons for the LA reconstruction were not explored due to the insufficiency of LR models in delineating through-plane data (Fig. 3).

#### Quantitative assessment

The quantitative significance of the artifacts on strain estimation was estimated through an analysis of the image quality metrics. The performance of SRR in reconstructing the images was determined using the structural similarity indices (SSIM) and root-mean-square errors (RMS). The metrics were calculated across all the SA and LA acquisitions for the SR models of $\mathrm {r_{1} = 0.5}$, against the LR models (Table 1). SR images with $\mathrm {r_{1} = 1}$ were compared to the original image acquisitions. Consistent with the qualitative assessment, *natural* interpolation models of SR with $\mathrm {r_{1} = 0.5}$ produced images of high structural similarity with the original SA stacks (SR-R $= 0.8526 \pm 0.1172$, and SR-O $= 0.8234 \pm 0.1187$). Regardless of the interpolation schemes, SSIM of above 90% was encountered only at the SA images of the apical slice and the apex. Despite a relative absence of artifacts in the SR-O images (Supplementary Fig. S2), higher levels of similarity were achieved using SR-R (Table 1). These SSIM values were likely to have stemmed from the similarity in blurring effects between the SR-R and LR images.

**Table 1:** Image quality metrics for super-resolution reconstruction (SRR) versus conventional reconstruction. The results are presented as mean ± standard deviation for the reconstruction of the original low-resolution (LR) eight SA and five LA image planes.Linear and Natural denote the nature of the global interpolation scheme. SSIM: structural similarity index, RMS: root-mean-square error. SR-O: SRR using the combination of SA and orthogonally sampled LA images; SR-R: SRR using the combination of SA and radially sampled LA images.

		Linear ($\hbox {r}_1 = 0.5$)	Natural ($\hbox {r}_1 = 1.0$)	Natural ($\hbox {r}_1 = 0.5$)
SR-R	SSIM	$0.7856\pm 0.1478$	$0.715 \pm 0.1356$	$0.8526\pm 0.1172$
	RMS	$0.1542\pm 0.0841$	$0.1851 \pm 0.1143$	$0.1556\pm 0.1087$
SR-O	SSIM	$0.7743\pm 0.1345$	$0.7045 \pm 0.1085$	$0.8234\pm 0.1006$
	RMS	$0.1523\pm 0.0846$	$0.2098 \pm 0.1119$	$0.1234\pm 0.1187$

### Validation of SRR in CMR using an in silico phantom

**Fig. 4:**
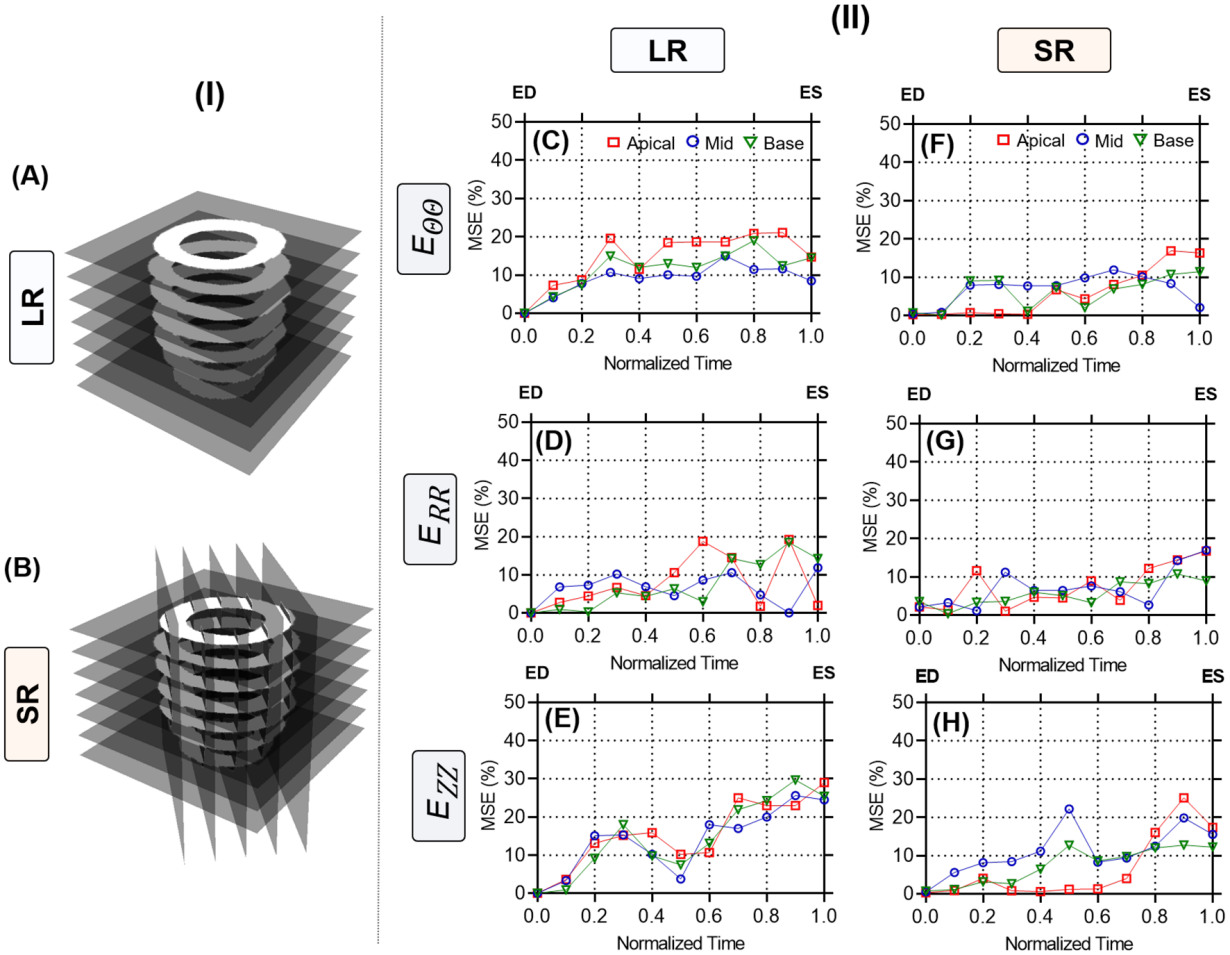
Mean squared error (MSE) curves comparing the errors in strain estimation between the image-derived and ground-truth strains established via an in-silico phantom at various timepoints between end-diastole (ED) and end-systole (ES). Strains were calculated through image registration using (**A**) low-resolution (LR) and (**B**) super-resolution (SR) phantom images. MSE curves for all three strain quantities are presented at three sectional planes of the LV derived using the (**C**-**E**) LR and (**F**-**H**) SR phantoms. $E_{\Theta \Theta }:$ circumferential, $E_{RR}:$ radial, and $E_{ZZ}:$ longitudinal strains.

The accuracy of the SRR framework in calculating myocardial strains was first validated using an in silico heart phantom. The ablation study presented greater MSE accuracy in a combined image stack of 8 SA and 5 LA images than 8 SA and 3 LA image stack (Supplementary Fig. S3), Subsequently, a thorough analysis was conducted at multiple SA slices by comparing SR phantoms generated using a stack of eight LR images and SRR using eight SA images and five orthogonally sampled LA images against LR phantoms (Figs. 4A and B). The accuracy of the respective stacks was estimated using MSE analyses, and readings are reported for the time-course progression of contractile strains described by both phantoms at the apical, mid, and basal planes (Figs. 4C-H). Both phantoms presented comparable errors in the estimation of end-systolic $E_{RR}$ (LR vs SR: MSE = $\mathrm {9.4033}$ vs. $\mathrm {10.0104}$). However, improvements were noted in the calculations of both $E_{\Theta \Theta }$ (LR vs SR: MSE = $\mathrm {12.5700}$ vs. $\mathrm {9.9553}$) and $E_{ZZ}$ (LR vs SR: MSE = $\mathrm {26.2933}$ vs. $\mathrm {15.0667}$) at ES. Additionally, improvements due to SRR were observed in the early stages of contraction for all three strain quantities (Normalized time: 0 - 0.4).

**Fig. 5:**
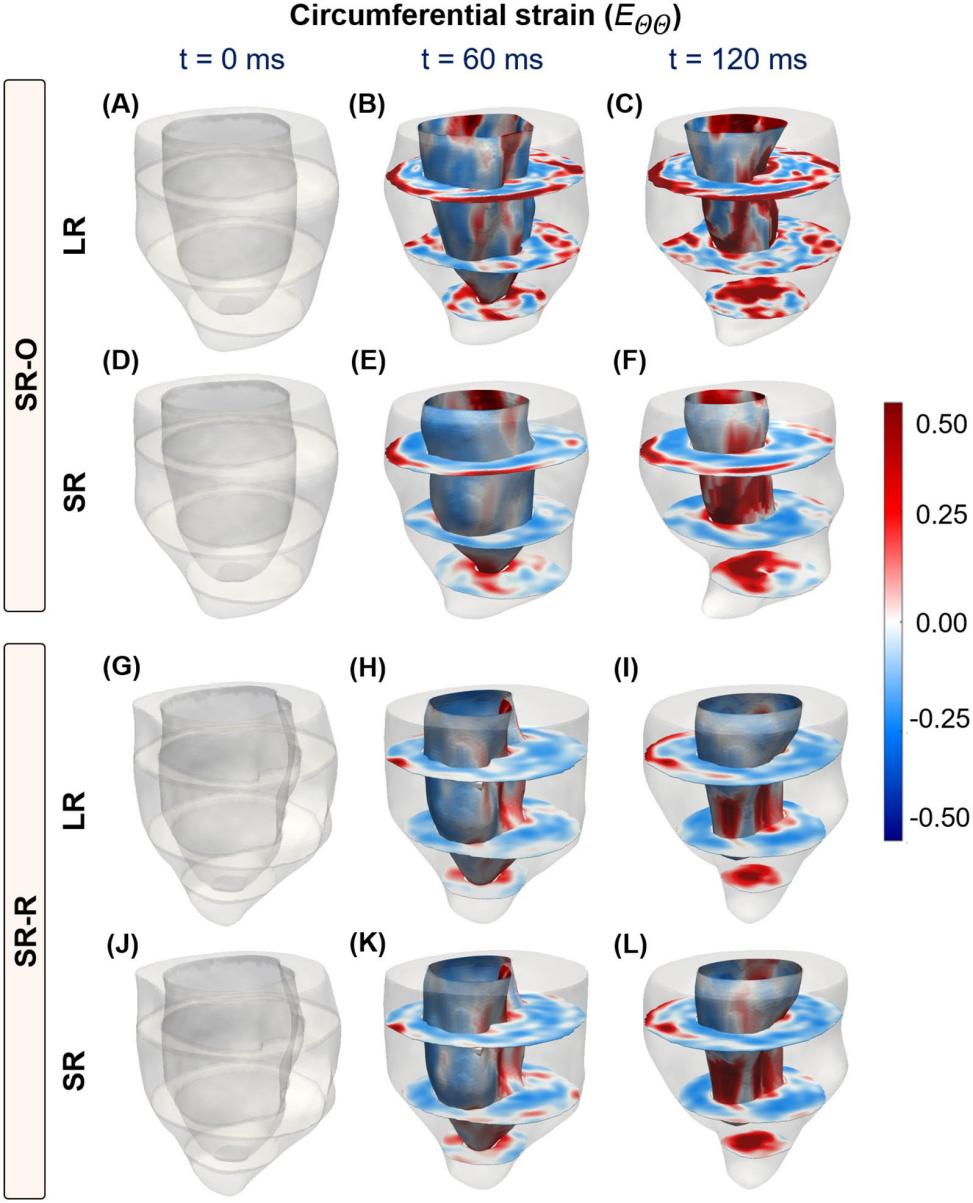
Time-course progression of circumferential strains from end-diastole (ED; $\mathrm t = 0$ ms) to end-systole (ES; ${\textrm{t}} = 120$ ms) at the basal, mid, and apical short-axis (SA) slices, and the endocardial wall of the LV. Strains are shown for super-resolution reconstruction (SRR) of the LV using two configurations: (**A**–**F**) SA images combined with orthogonally sampled long-axis (LA) images (SR-O), and (**G**–**L**) SA images combined with radially sampled LA images (SR-R). (**A**–**C**, **G**–**I**) Strain calculations are also presented for LV volumes reconstructed using the corresponding low-resolution image stacks.

**Fig. 6:**
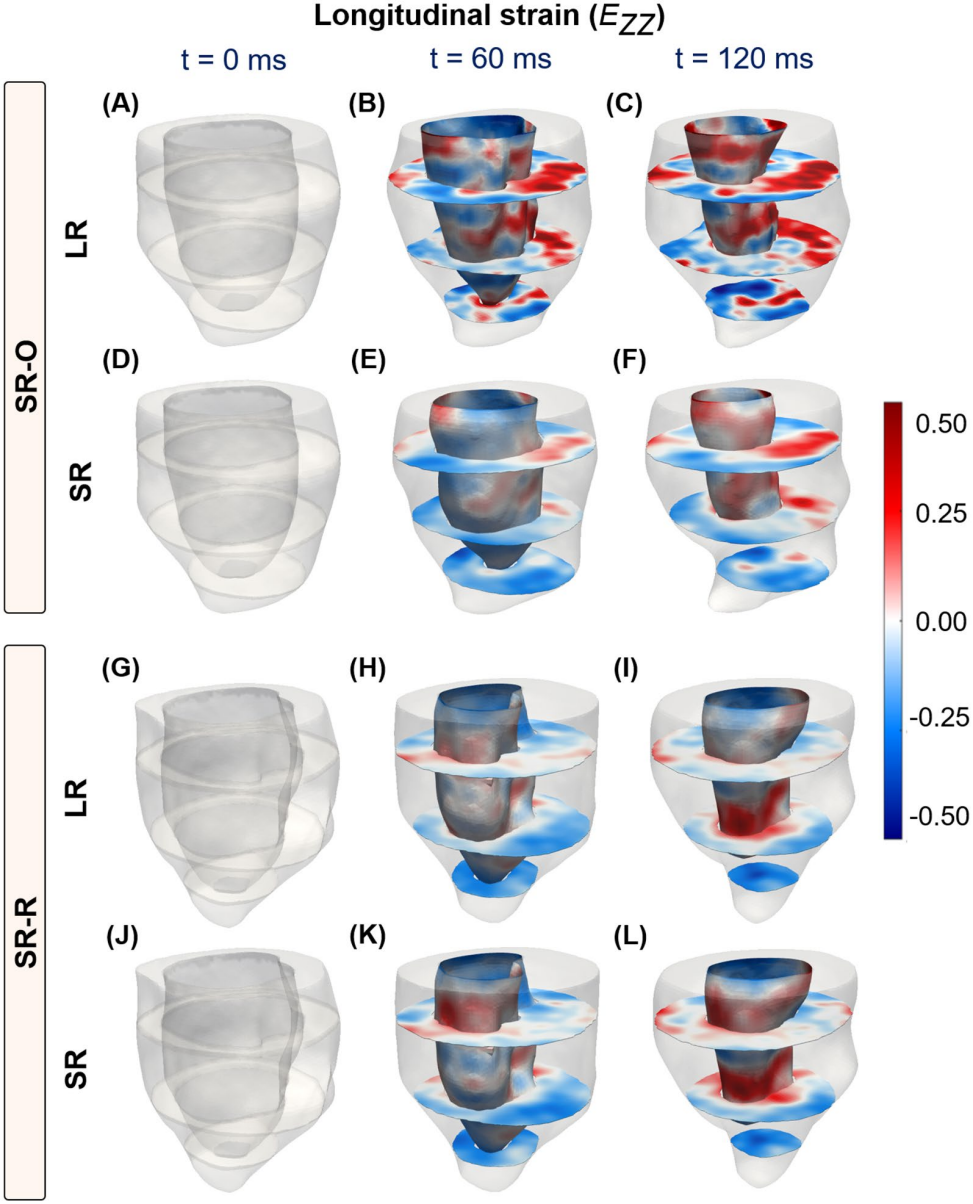
Time-course progression of longitudinal strains from end-diastole (ED; ${\textrm{t}} = 0$ ms) to end-systole (ES; ${\textrm{t}} = 120$ ms) at the basal, mid, and apical short-axis (SA) slices, and the endocardial wall of the LV. Strains are shown for super-resolution reconstruction (SRR) of the LV using two configurations: (**A**–**F**) SA images combined with orthogonally sampled long-axis (LA) images (SR-O), and (**G**–**L**) SA images combined with radially sampled LA images (SR-R). (**A**–**C**, **G**–**I**) Strain calculations are also presented for LV volumes reconstructed using the corresponding low-resolution image stacks.

### 4D myocardial strain distribution in the murine heart

#### SRR suppresses unrealistic strain estimation

Mouse-specific 4D strains were calculated using the SR and LR models at various time points describing the cardiac cycle, and a summary of arbitrary SA and LA images describing the cardiac cycle is presented in the supplementary material (Supplementary Figs. S4 and S5). The resulting strain maps are reported for both the diabetic (Figs. 5-6 and Supplementary Fig. S6) and WT mice (Supplementary Fig. S7) In contrast to the conventional LV models, transmural and regional variations in strains were quantified over the entire LV using SRR (Fig. 6 and Supplementary Fig. S6). While strains have been reported for both models, strains for the conventionally reconstructed LV were merely mapped onto the SR models using a nearest neighbor approximation (Figs. 5A-C, G-I and 6A-C, G-I). A visualization of the epicardial strains has been presented using only the SR models, as the LR models revealed unrealistic through-plane strains. As per literature^33,34^, any strain over a limit of 50% was designated to be non-physical. At ES, regional epicardial strains using SR showed varying degrees of contraction between the base and the apex, with apical shortening exceeding the other regions (Supplementary Fig. S6). Clinically relevant estimates of strains are often measured at the basal, mid, and apical slices. Accordingly, the circumferential (Fig. 5) and longitudinal (Fig. 6) strains were visualized at these slices and along the endocardium. Although qualitative similarity in strain distribution was observed between the two methodologies, there was a significant difference in the peak strains. Specifically, for the LR model, there was a significant presence of positive longitudinal strains despite global LV shortening (Septal; LR, $E_{ZZ} = 0.7342$ vs SR-O, $E_{ZZ} = 0.4985$. Lateral; LR, $E_{ZZ} = 0.5146$ vs SR-O $E_{ZZ} = 0.3126$). Also, the regional circumferential shortening seen in SR was recapitulated in the LR model with similar differences in peaks. However, there was an abundance of non-physical transmural strains, which were suppressed by both SR-O and SR-R models (Figs. 5C, F). The effects of these artifacts were mitigated in the AHA segmentation plots, where each strain value was visualized as the average of the estimated strains within a segment (Supplementary Figs. S8 and S9).

**Fig. 7:**
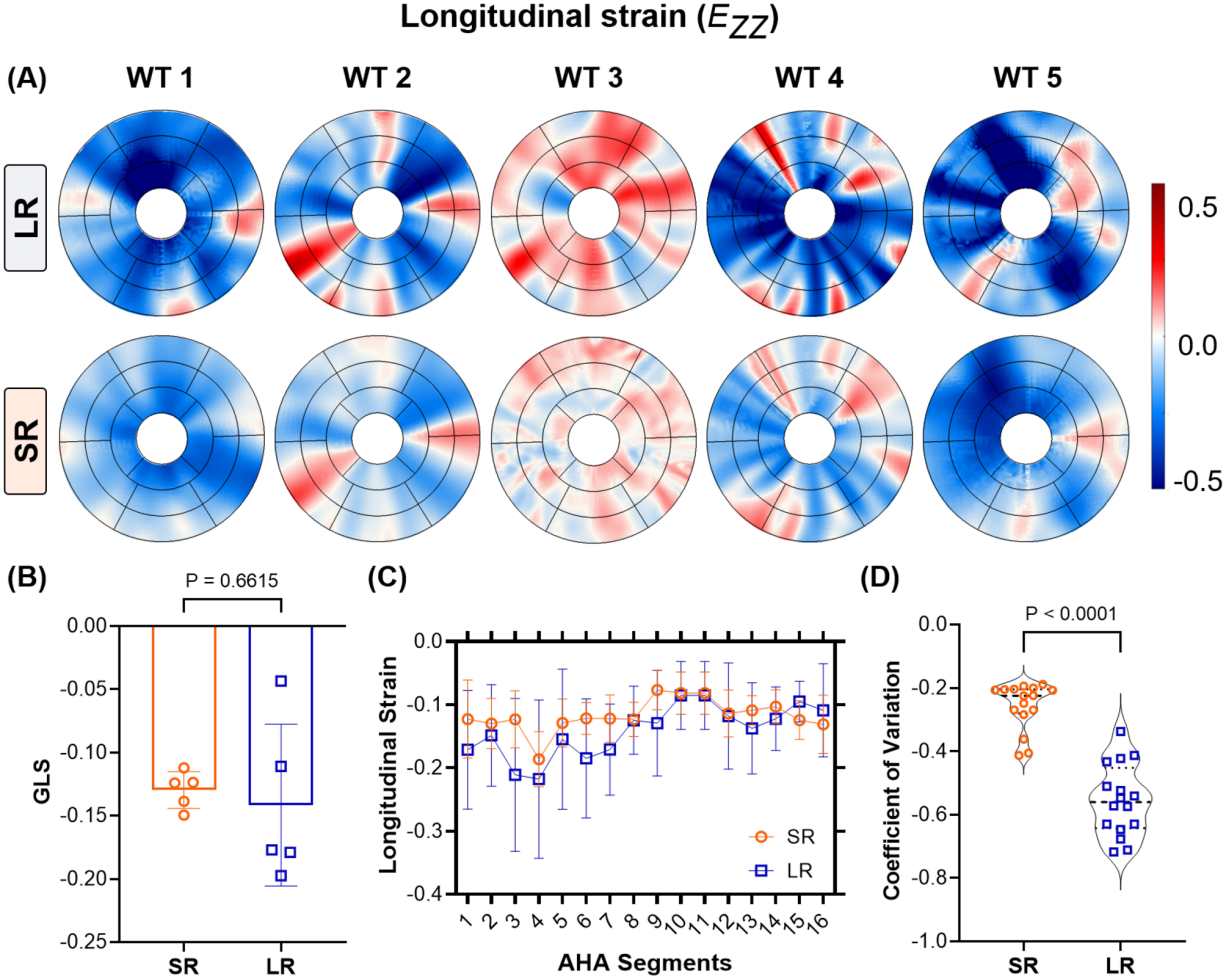
Evaluation of the spatial homogeneity of myocardial strain distribution using low- and super-resolution (LR and SR, respectively) imaging in wild-type (WT) mice (n = 5). (**A**) Regional distribution of end-systolic longitudinal strain, presented in AHA segmentation plots at the base, mid, and apical slices. (**B**) Estimation of global longitudinal strain (GLS) as the average of all regional strain quantities in each AHA segment. (**C**) Comparison of the average longitudinal strain in each AHA segment, with the values represented as mean ± standard deviation (SD). (**D**) Violin plots of the coefficient of variation (CV) of the strain distribution at each segment for all mice.

#### SRR improves the spatial and intra-cohort homogeneity of myocardial strains

The time-course homogeneity in regional myocardial strain distribution estimated between the SR-O and SR-R images and corresponding LR images are reported. As opposed to the considerable variations in strain patterns that were noted between the two LR models (Figs. 5C, I and 6C, I), the SR images provided more consistent strain distribution between both acquisition strategies. Variations in the regional contractile patterns were observed between the SR and LR models. For instance, large areas of circumferential thickening, concentrated in the lateral regions, were removed in the SR-O models, with the mean strain in all three sections of the LV reducing considerably (Lateral; LR, $E_{\Theta \Theta } = -0.0870 \pm 0.0140$ vs. SR, $E_{\Theta \Theta } = -0.1541 \pm 0.0277$). There was a great agreement in the contractile patterns between SR-O and SR-R, with the lateral regions of the LV showing similar contractile patterns indicating improvements in reproducibility (Lateral; SR-O, $E_{\Theta \Theta } = -0.0870 \pm 0.0140$ vs. SR-R, $E_{\Theta \Theta } = -0.07124 \pm 0.0148$).Moreover, intra-cohort strain variability investigated as the degree of spatial heterogeneity in the regional strain distribution is reported for five WT mice (Fig. 7). The suppression in unrealistic strain patterns was observed across all mice through the SR models (Fig. 7A), and estimations of GLS for conventional (LR) strain calculations tended to show higher variability across five mice compared to that of SR measurements (Fig. 7B). Similarly, SR demonstrated considerable improvements in minimizing the standard deviation (SD) at each segment between the mice (Fig. 7C), with a 50% reduction in the coefficient of variation measured as the ratio of the SD to the mean at each segment (Fig. 7D; SR vs LR: −0.2186 vs. −0.5732).

## Discussion

### Enhanced image acquisition facilitated 4D strains in small animal CMR

Most traditional CMR-based methods of motion tracking, including tagging, are often tested and validated using human-specific images of the LV. Apart from the well-established differences in physiology between mice and humans, the rapid heart rate of mice compromises image quality and is a significant source of variability between operators, machines, and subjects^15,35^. In particular, 4D strains are influenced primarily by substandard delineation of in-plane cardiac motion and insufficient data for through-plane measurements^36^. These limitations have contributed to facile 2D in-plane representations of the complex LV motion. While some strategies have extrapolated longitudinal deformation by interpreting deformation patterns at the endocardial and epicardial layers^12^, others have used contrast-enhanced image acquisitions to assess regional distributions of 3D strains in SA and LA images^37^. An ablation study was first conducted to identify the ideal combination of SA and LA images to accurately capture mouse-specific 4D strains using standard cine CMR (Supplementary Fig. S3). The in-silico phantom ensures a strong 4D kinematic benchmark, which facilitates the analysis of regional strain accuracy^15^. SRR-derived strains were validated by using an in-silico phantom, and SRR was noted to improve the description of through-plane motion. Additionally, the combination of 8 SA and 5 LA images revealed mean-squared errors of under 10%, and thus was used as the reference strategy for mouse-specific imaging (Fig. 2). Subsequently, a thorough analysis of image resolution and comparisons with conventional LR images that exhibit significant through-plane anisotropy revealed marked differences in the estimation of in- and through-plane strains (Figs. 5-6 and Supplementary figs. S8-S9).

### SRR in CMR improved intra-cohort strain homogeneity in mice

The LV exhibits twisting during systolic contraction, resulting in significant through-plane deformation with corresponding in-plane mechanical adjustments via radial thickening and circumferential shortening. These deformations translate as “out-of-plane” pixel motions that are not captured by traditional CMR imaging of the LV. Standard functional imaging is often limited to 7-9 SA planes with an optional LA or four-chamber view. The resulting pixel set is heavily anisotropic, which impedes accurate strain calculations. We hypothesized that an SRR-based methodology combining scan planes of varying orientations would considerably reduce pixel spacing, thereby improving strain calculations. An in-silico ablation study was conducted to determine the best combination of LA and SA images (Supplementary Fig. S3) and applied to a cohort of WT mice. In the evaluation of longitudinal strains, a significant presence of positive regional strains was noted in the LR model (Fig. 6 and Supplementary Fig. S7) despite similar global shortening (Fig. 7). Notably, the LV was shown to elongate at ES when strains were generated using the SA stack (Figs. 6C, I). These observations were expected as the through-plane anisotropy distorts displacement derivatives and subsequent strains (Eq. 8). The interpolation module of the SRR framework was specifically designed to address this concern. Piecewise cubic interpolants have been shown to be effective in reconstructing 2D image planes from non-uniform datasets^38^. The *natural* interpolation method showed very minute RMS errors and high SSIM in reconstructing individual image planes (Table 1). Despite significant upsampling of query points in all directions, average SSIM values of 80% were still achieved with very minimal artifacts (Appendix; Table A1). Building on these minor deviations in feature preservation, the pixel spacing using SRR was increased to about 25 times the original acquisition. Through this nearly isotropic pixel set, apical and endocardial shortening was captured more accurately in both SRR models at ES (Figs. 6F,L), with a very minor presence of positive longitudinal strains across all WT mice (Fig. 7). The benefits of SRR in improving longitudinal shortening were validated using the in-silico phantom while minimizing errors at three different planes of the LV. These errors can be attributed to motion artifacts, especially at the tissue-pool interface of the endocardium, that confound image registration. Despite the presence of these artifacts, errors in all strain quantities were restricted to under 10%, highlighting the potential improvements in 4D strain calculations (Supplementary Data S1).

### Strain reproducibility was enhanced by reducing image sharpness via SRR

The standardization of regional strain calculations using any imaging modality is challenged by inter-study and inter-vendor variability. These limitations are either caused by assumptions in delineating cardiac motion, which differ between calculation strategies, or, in most cases, are dependent on the operator’s capabilities. The inadequacy of temporal resolution in standard CMR images results in sharp gradient changes between time points, thereby producing errors in registering consecutive image frames. A strong correlation was noted between sharp image gradients and the generation of non-physical strains, which necessitate regularization. A consequence of regularizing the image registration process was the underestimation of strains, especially at the tissue-pool interface of the endocardium. In the absence of this regularization, notable variations were observed between the two LR models of 4D strains with pronounced differences in both the peak values and the patterns (Figs. 5C, I, and 6C, I). The images on the second day (SR-R) were noticeably blurred compared to the first (Supplementary Fig. S2A and I). resulting in a disparity in endocardial sharpness and subsequent strain estimation. By defining a convex hull of pixels with boundaries at the endocardium, the edge sharpness was increased using SRR, and the chamber pixels were isolated from the myocardial tissue. The diffusion of gradients across the edges was thus inhibited, and the effects were translated as such in the strain calculations (Equations 6 and 8). Given the minimal presence of artifacts in the SR-O images, the protocol was implemented in the study homogeneity of 4D strains in a cohort of WT mice. As opposed to the significant deviation in strains derived from the LR model, similar contraction patterns were observed between the different WT mice using the SR images (Fig. 7), indicating a pathway toward the reproducibility of 4D regional strains. However, contrary to the improvements in transmural strain accuracy, calculations at the endocardial and epicardial borders can still be improved since the corresponding pixels were subjected to regularization through radial basis functions to maintain continuity in the global interpolation scheme at the tissue-pool interface.

### Potential of 4D strains for prognostic assessment of the myocardium

Heart diseases such as myocardial infarction and hypertrophic cardiomyopathy leading to heart failure are clinically diagnosed through global and regional volumetric and functional indices. Measurements of diastolic and systolic LV elastances, as well as diffusion tensor imaging and histological analyses, have confirmed mechanical and architectural changes in the LV due to a variety of cardiac remodeling mechanisms. These myocardial adaptations lead to alterations in cardiac motion and can be detected by quantifying myocardial strains. In cases of radiation therapy-induced cardiotoxicity, functional impairments are not immediately apparent through EF measurements. In the absence of worsening EF, regional strain calculations have been shown to be sensitive markers, especially at an early stage^6,39^. However, the innate complexity of regional cardiac motion, resulting from intricate myocardial architecture and passive and active phases of motion^40^, remains to be a prominent challenge in rigorously quantifying full 4D cardiac motion in vivo. Image-based strain models usually restricted to 2D planes often yield confounded representations of the resulting myocardial deformation. The complex nature of 4D ventricular kinematics^41^ demands the establishment of a standardized quantification methodology. The regional strain analysis presented herein carries the potential to establish a reproducible 4D representation of myocardial deformation. Through the SRR framework presented in this study, significant improvements in image quality, and strain calculations were achieved. This rigorous quantification of 4D strains can further be used in conjunction with real-time volumetric, and hemodynamic readings to estimate the intrinsic material properties of the myocardium^42^. Thus, a multiscale mathematical approach comprising organ-level measurements, and tissue-level mechanical characteristics can be posed to assist in clinical intervention.

### Limitations

Despite the proposed utility of the SRR framework in promoting the prognostic efficacy of 4D myocardial strains, there exist a few limitations. First, investigations into the intra-cohort variability of 4D contractile patterns were restricted to WT mice. While this allowed us to present comprehensive image reconstruction and 4D strain reproducibility within controlled conditions, the implementation of the SRR framework in additional subjects is surely needed to further evaluate its performance and optimize its application across different pathophysiological conditions. Second, we were limited by the imaging capabilities in acquiring HR scans as a ground-truth metric to evaluate the performance of SRR in image reconstruction. However, this is common in most SRR methods^22,43^ as capturing these acquisitions is limited by factors such as hardware capabilities (e.g, limited scan speeds) and government regulations. Finally, the SRR framework was susceptible to the generation of artifacts during image-plane reconstruction. Currently, the SRR framework relies on standard scattered data interpolation to generate HR images. Since the HR images were generated through interpolation, we noted artifacts in the form of discontinuous straight lines across the SA slices. Crucially, we suspect these artifacts to have influenced the derivation of the image gradients and, subsequently, the strain estimation. We expect these artifacts to be reduced by implementing deterministic interpolation schemes^44,45^, yet to be implemented in MRI scans, or by using physics-informed image registration algorithms^46^. Alternatively, individual slices could be subjected to edge-enhancing diffusion filters. These filters have been known to reduce artifacts in image reconstruction by impeding diffusion across edges and have also been implemented in MRI scans of the brain^47^.

## Conclusion

In this study, we presented a novel and comprehensive analysis of 4D myocardial deformation through the SRR in CMR framework. The framework offers a translational tool that leverages combined CMR acquisitions and tissue classification strategies to improve LV reconstruction with minimal noise and artifacts. A detailed representation of 4D myocardial strains was made possible with such analysis, offering the potential to establish a continuous 4D LV structure-function relationship as a standard practice. To the best of our knowledge, this is the first application of any SRR methodology to enhance the in-vivo characterization of soft-tissue mechanics.

## Supplementary Information

Supplementary Information.

## Data Availability

Code and supporting data, including animations and results, are available in the ’SRR in CMR’ Github repository (https://github.com/Tanmay24Mukh/SRR_in_CMR.git). Any additional data that support the findings of this study are available from the corresponding author, R.A., upon request.
