# Supplementary material for: Complete spatiotemporal quantification of cardiac motion in mice through multi-view magnetic resonance imaging and super-resolution reconstruction
Source: Sci Rep. 2025 Aug 13;15:29696. doi: 10.1038/s41598-025-11766-5 (PMC12350669; doi:10.1038/s41598-025-11766-5)
Supplement: Supplementary file 1 — Supplementary Information. [file 41598_2025_11766_MOESM1_ESM.pdf]

Supplementary material

Appendix

A1 Image quality analysis

Prior to strain estimation, the quality of the SR-O and SR-R images was assessed to evaluate the image reconstruction performance of SRR. Strain calculations have been observed to be sensitive to the quality of individual image planes, with artifacts such as discontinuities and blurring affecting the diffeomorphic demons algorithm. Specifically, the spatial definition (image sharpness) of the endocardial and epicardial borders in the reconstructed images was identified as a major contributor to strain reproducibility. Here, reproducibility refers to qualitative and quantitative similarities in strain patterns for the study on different days of image acquisition. The overall effects of each entity on strain estimation were grouped into three categories, (i) the endocardial sharpness, (ii) the epicardial sharpness, and (iii) artifact presence. Here, sharpness refers to the definition of the myocardial tissue at each region and was evaluated using image acutance, which is a subjective metric that relates the spatial resolution of an image to its spatial frequency. A small interrogation window describing the endocardium and epicardium was sampled, and acutance was evaluated using the image gradients such that:

$$D = \Sigma_1^n \nabla I = \Sigma_1^n \begin{bmatrix} \nabla_x I \\ \nabla_y I \end{bmatrix}, \tag{A1.1}$$

where **D** is the acutance, **I** is the interrogation window, and ∇_x and ∇_y are the gradient operators in the x and y directions of the window. Mean acutance was evaluated using the root-mean-squared (RMS) value of all the image gradients. A manual qualitative analysis in terms of a 5-point Likert scale (1 to 5) was performed for the SR images generated using the first imaging protocol, with each value signifying the levels of strain reproducibility. The lowest score denotes abject variability, and the highest indicates completely reproducible strains. The scoring scale for categories (i) and (ii) ranges between 1 = no distinct border due to poor acutance and 5 = perfectly defined border or high acutance. Scoring for artifact presence was determined between 1 = significant presence of unnatural features such as gradient discontinuities in the form of lines or the isolated presence of sharp pixels and 5 = no artifacts.

The quality of SR-O and SR-R in reconstructing the eight original LR SA images was evaluated, and Likert scores were assigned based on the mean acutance values for both SR models generated using the *linear* and *natural* interpolation schemes. Results are also reported for their corresponding conventionally reconstructed models (Table A1).

Table A1. Qualitative analysis of SRR performance over a 5-point Likert scale. The original eight SA image planes were considered. All the results are presented as mean ± SD. Linear and Natural denote the nature of the global interpolation scheme. SR-O: the combination of SA and orthogonally sampled LA images; SR-R: the combination of SA and radially sampled LA images

		**LR**	**Linear** ($\mathbf{r}_1 = 0.5$)	**Natural** ($\mathbf{r}_1 = 1.0$)	**Natural** ($\mathbf{r}_1 = 0.5$)
**SR-R**	Epicardium	4.12 ± 0.54	4.20 ± 0.21	3.87 ± 0.15	4.43 ± 0.23
	Endocardium	3.73 ± 0.31	4.00 ± 0.51	3.53 ± 0.56	4.31 ± 0.34
	Artifacts	4.40 ± 0.27	4.14 ± 0.24	2.40 ± 0.46	3.97 ± 0.38
**SR-O**	Epicardium	4.32 ± 0.18	4.40 ± 0.12	3.88 ± 0.21	4.46 ± 0.13
	Endocardium	4.06 ± 0.16	4.11 ± 0.14	2.64 ± 0.28	4.45 ± 0.12
	Artifacts	4.6 ± 0.18	4.23 ± 0.09	2.51 ± 0.17	4.08 ± 0.16

S1 Videos

Code and supporting data, including animations and results, are available in the 'SRR in CMR' Github repository (https://github.com/Tanmay24Mukh/SRR_in_CMR.git).

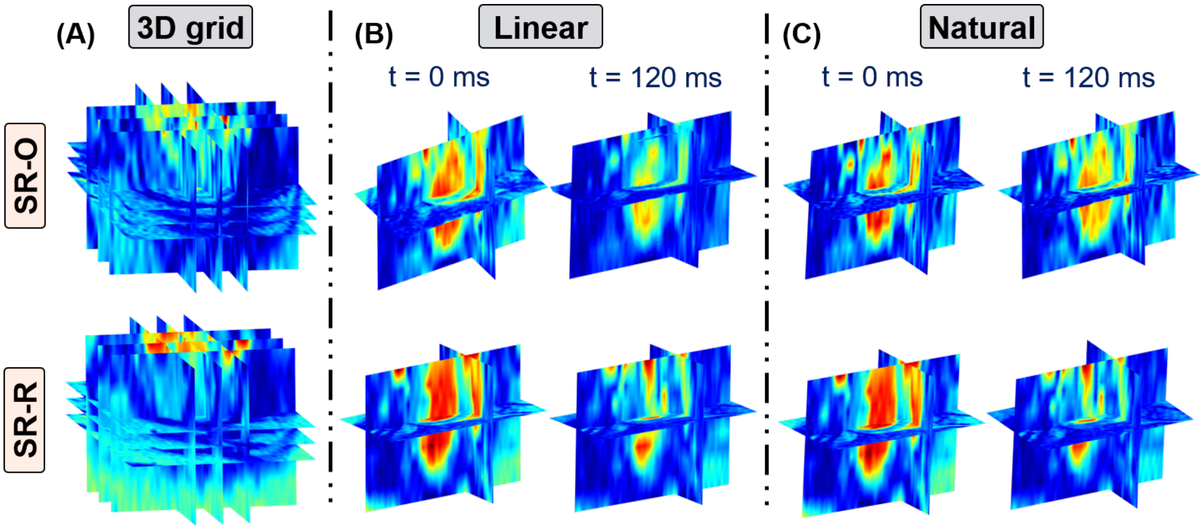

Supplementary Figure S1. (A) Representative super-resolution reconstruction (SRR) of the left ventricle (LV). The reconstructed LV is shown over arbitrary slices with the highest pixel intensities denoting blood. Representation of the cardiac cycle from (B) end-diastole (ED; $t = 0$ms) to (C) end-systole (ES; $t = 120$ms) over three orthogonal planes reconstructed using (top) SR-O and (bottom) SR-R. The SR grid was defined for a pixel spacing of 0.1 x 0.1 x 0.2 mm^3 corresponding to a resampling ratio of $r_1 = 0.5$. SR-O: SRR using the combination of SA and orthogonally sampled LA images; SR-R: SRR using the combination of SA and radially sampled LA images.

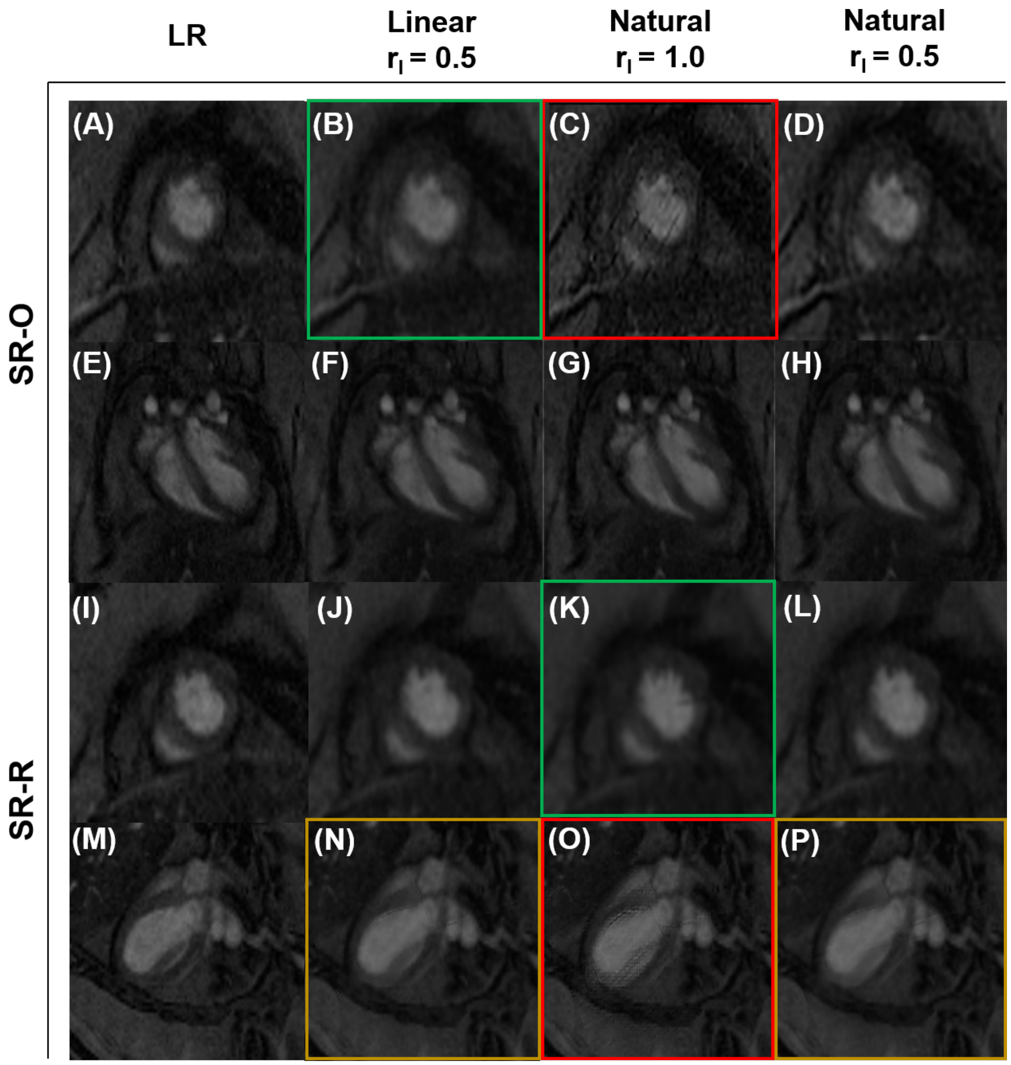

Supplementary Figure S2. Super-resolution reconstruction (SRR) of the original low-resolution (LR) image planes of (A-D, I-L) the mid short-axis (SA) slice, and (E-H, M-P) an arbitrary long-axis (LA) slice. Blurring and gradient discontinuities were observed with $r_l = 1.0$ contributing to the majority of the artifacts. Reconstructed image plane with the presence — blurring, — discontinuity artifacts, and — blurring & discontinuities. SR-O: SRR using the combination of SA and orthogonally sampled LA images; SR-R: SRR using the combination of SA and radially sampled LA images.

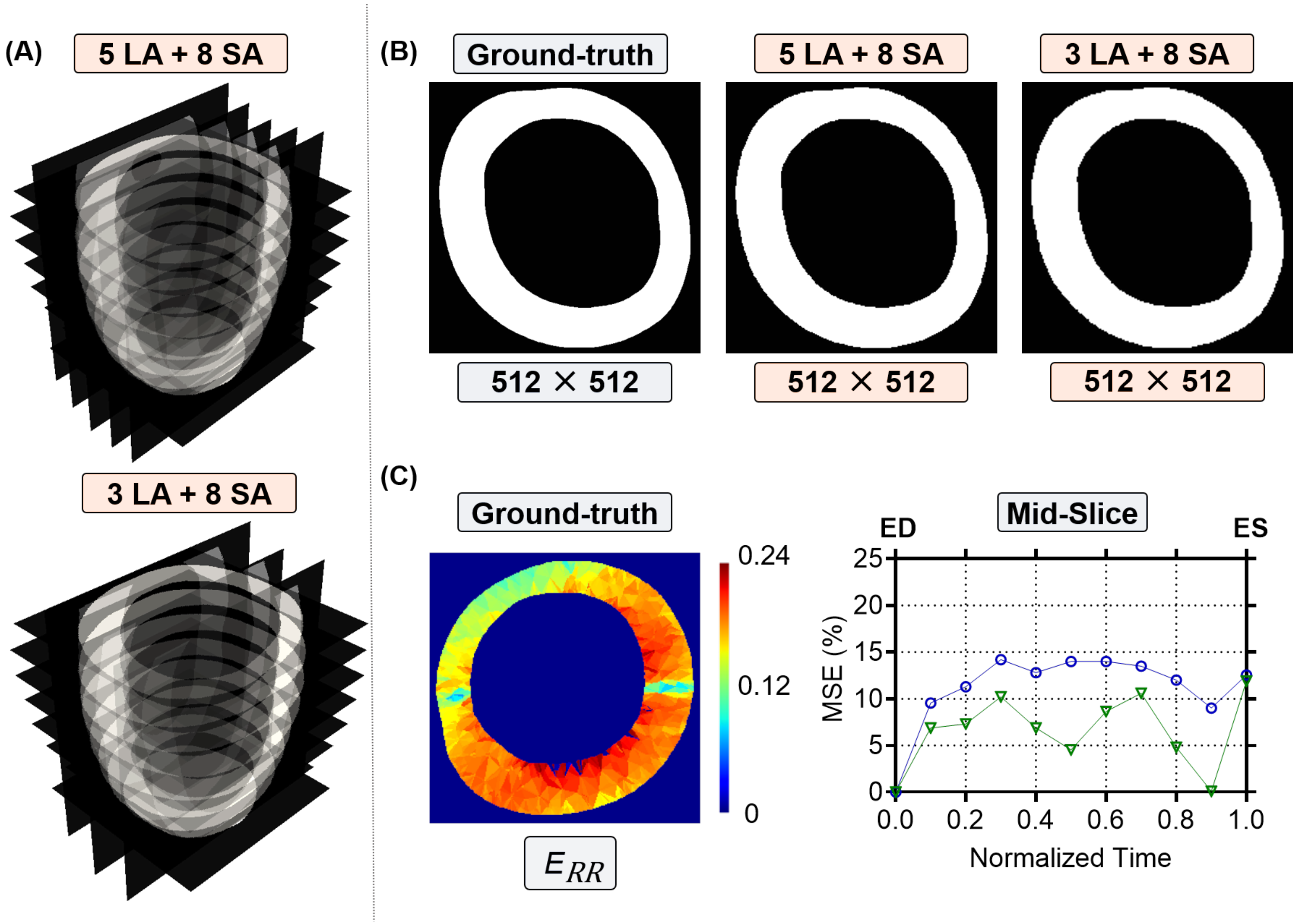

Supplementary Figure S3. In-silico ablation study to determine optimal combination of short- and long-axis (SA and LA) images to capture cardiac strains (A) Combination of eight short-axis (SA) image planes between the basal slice and the apex of the left ventricle (LV) combined with (top) five (5 LA + 8 SA) and (bottom) three long-axis (3 LA + 8 SA) slices sampled (top) orthogonally between the anterior and inferior walls of the LV on the first day. (B) Comparison of ground-truth high-resolution image and super-resolution reconstruction of the mid-SA slice using both 5 LA + 8 SA and 3 LA + 8 SA phantoms. (C) Mean squared error (MSE) curves comparing the errors in radial strain estimation between the image-derived and ground-truth strains at end-systole for both combinations of phantoms. Ground truth was established via an in-silico phantom at various time points between end-diastole (ED) and end-systole (ES).

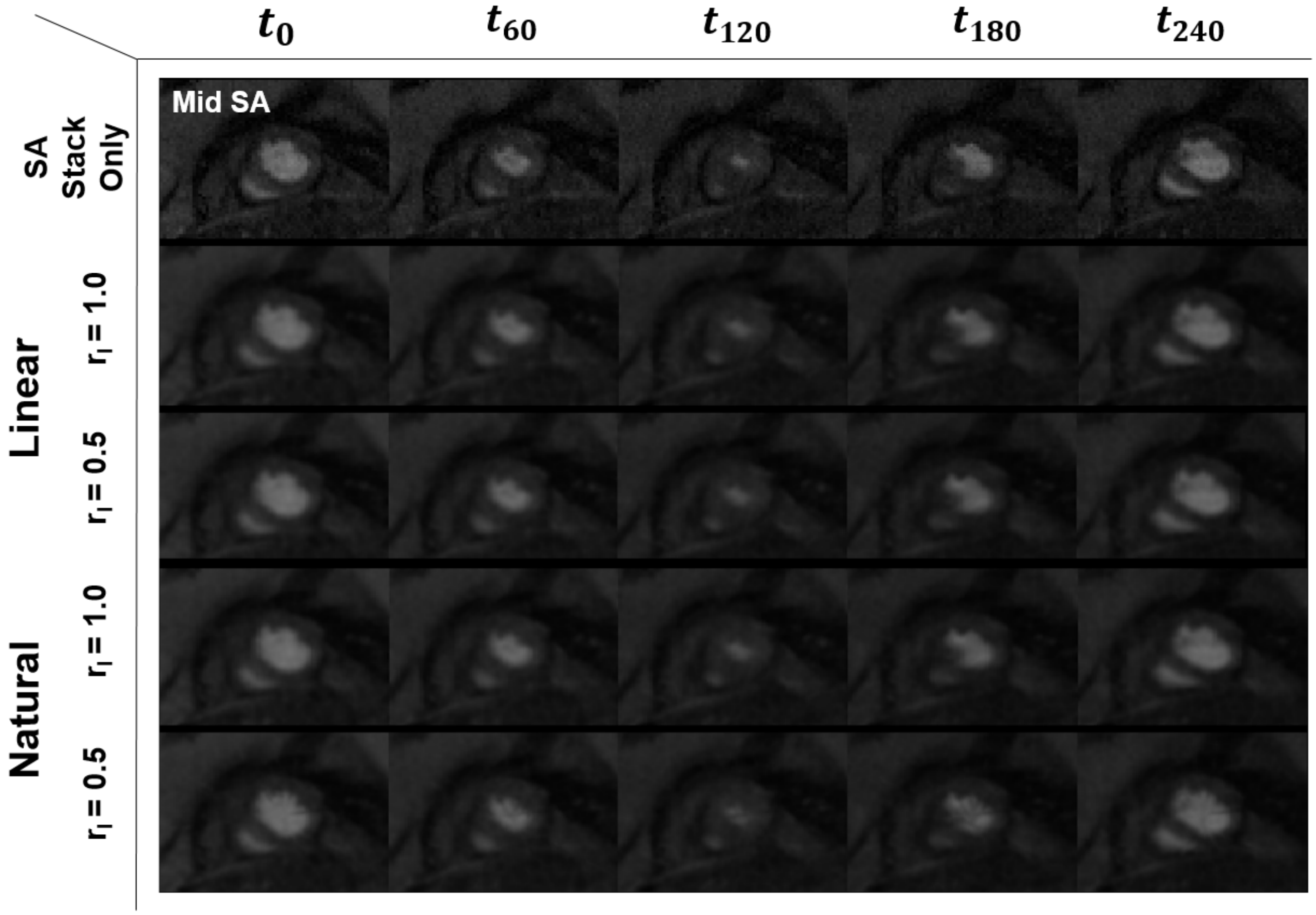

Supplementary Figure S4. Super-resolution reconstruction (SRR) of the mid-short-axis plane of a murine heart using the combination of SA and orthogonally sampled LA images. Images are shown for select timepoints within a cardiac cycle from ED to ES to ED with the total duration of t = 240ms. SA stack only represents the original low-resolution acquisitions, with Linear and Natural denoting the nature of the global interpolation. SR-O: the combination of SA and orthogonally sampled LA images; r_1: resampling ratio.

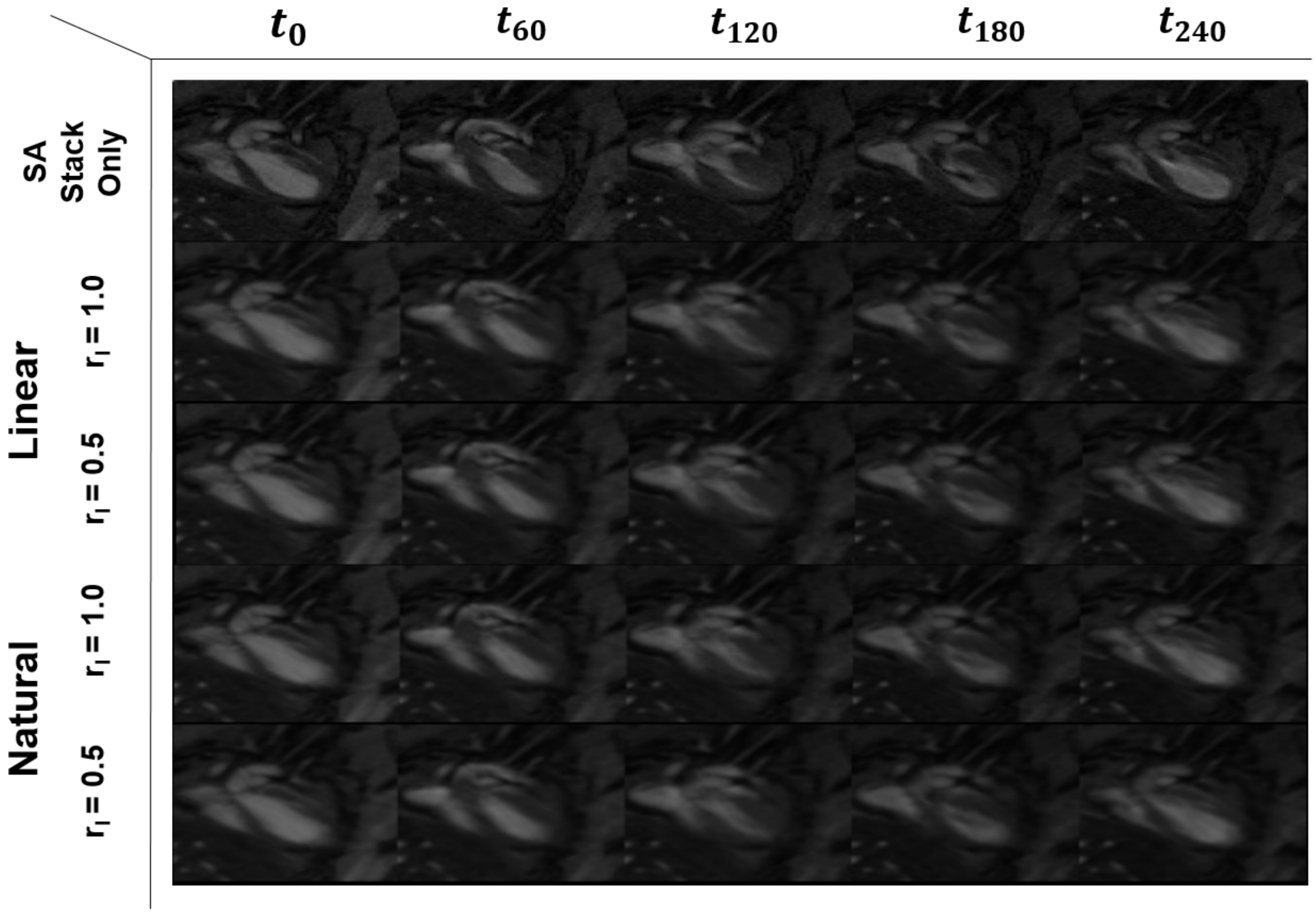

Supplementary Figure S5. Super-resolution reconstruction of an arbitrary long-axis plane of a murine heart using the combination of SA and orthogonally sampled LA images. Images are shown for select timepoints within a cardiac cycle from ED to ES to ED with the total duration of t = 240ms. SA stack only represents the original low-resolution acquisitions, with Linear and Natural denoting the nature of the global interpolation. SR-O: the combination of SA and orthogonally sampled LA images; r_1: resampling ratio.

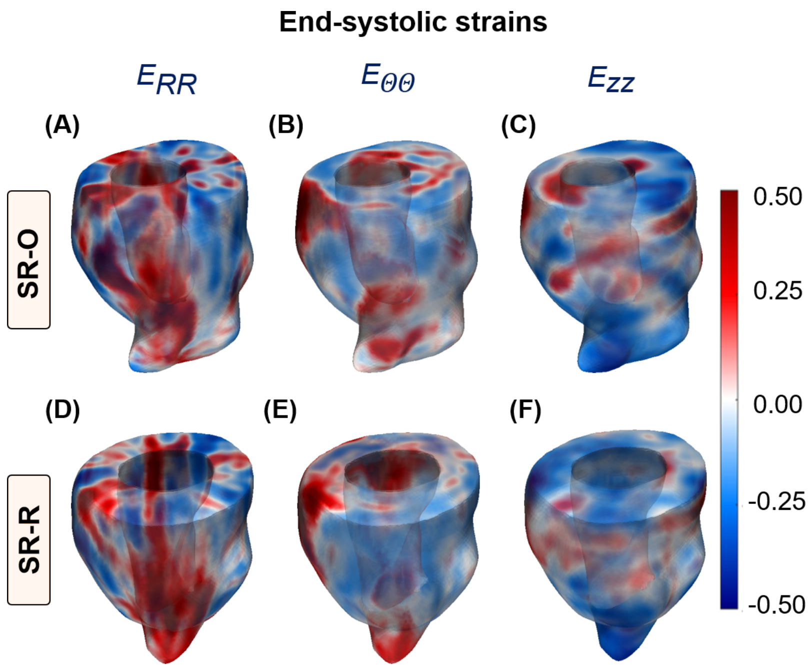

Supplementary Figure S6. Myocardial strains along the radial (E_{RR}), circumferential ($E_{\Theta\Theta}$) and longitudinal (E_{ZZ}) directions of the LV for (top) SR-O, and (bottom) SR-R at ES. Strains were mapped onto a reconstructed LV with a significant presence of positive radial deformation accompanied by regional distribution of negative strains in the circumferential and longitudinal directions in both reconstructions. SR-O: the combination of SA and orthogonally sampled LA images; SR-R: the combination of SA and radially sampled LA images. LR: low-resolution, SR: super-resolution

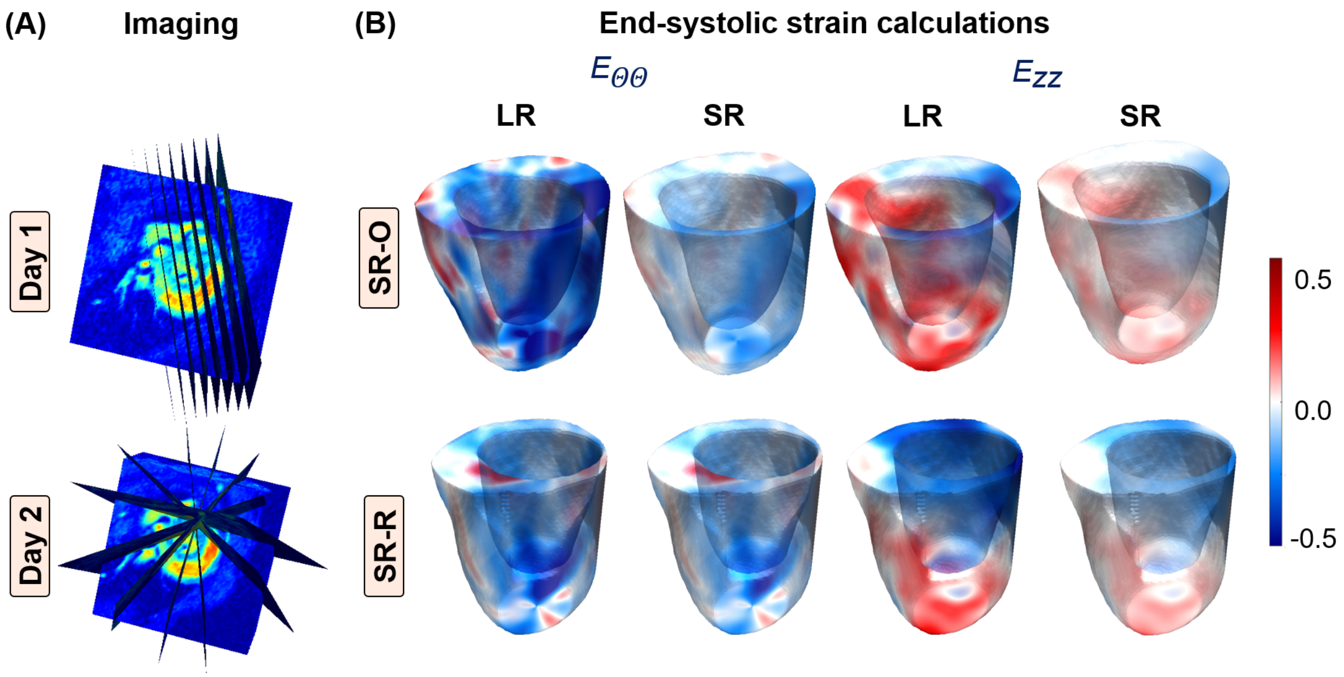

Supplementary Figure S7. (A) Imaging protocols used in the acquisition of cine-CMR images for a three-month-old wild-type (WT) mouse over a period of two consecutive days. Combination of eight short-axis (SA) image planes between the basal slice and the apex of the left ventricle (LV) combined with five long-axis (LA) slices sampled (top) orthogonally between the anterior and inferior walls of the LV on the first day and (bottom) radially about the LV chamber on the next day. (B) Myocardial strains calculated for the same WT mouse along the circumferential ($E_{\Theta\Theta}$) and longitudinal (E_{ZZ}) directions of the LV from CMR images acquired on (top) Day 1 and (bottom) Day 2 at end-systole. SR-O and SR-R: Super-resolution using orthogonal and radial LA images, respectively. LR: low resolution

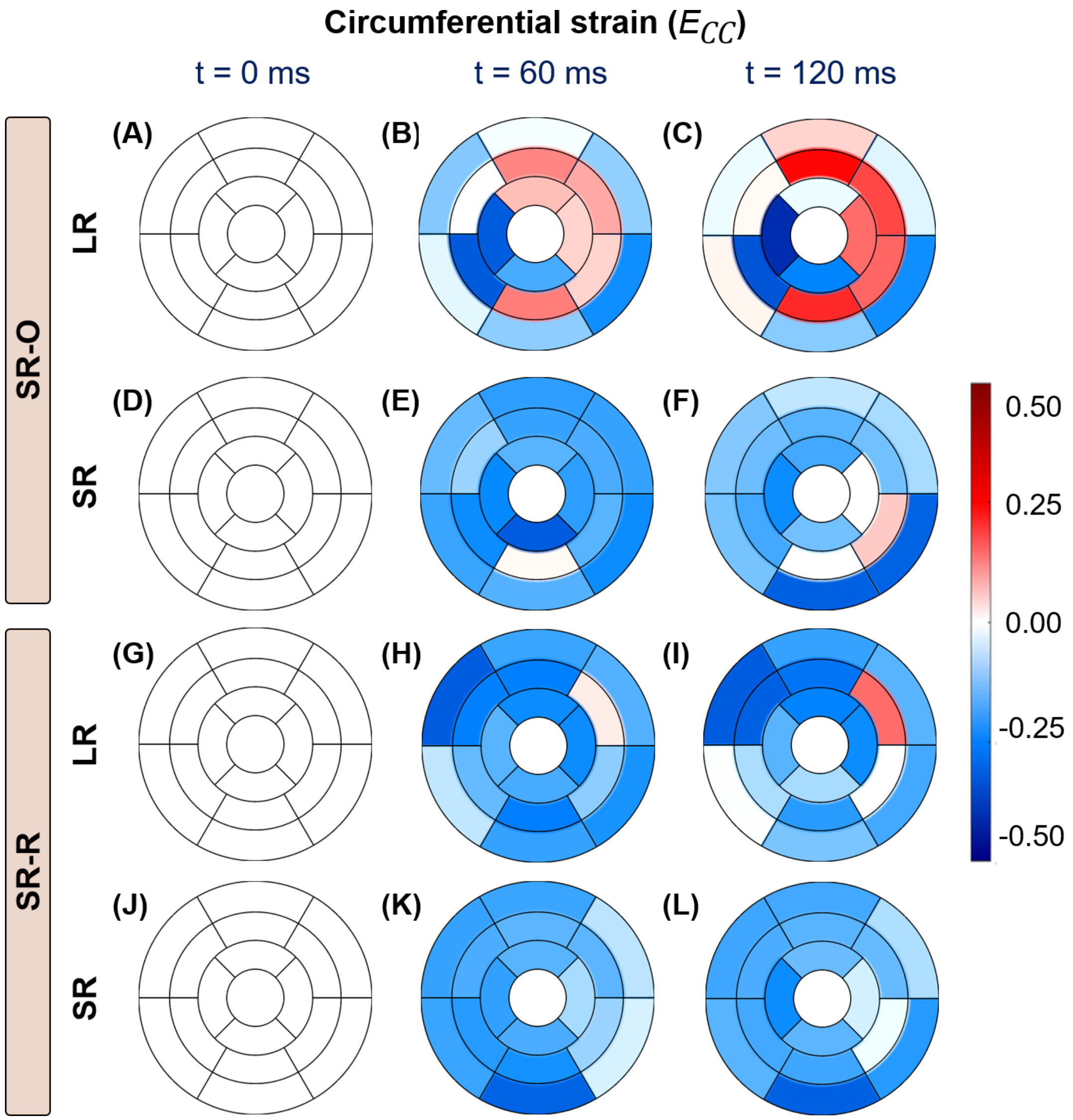

Supplementary Figure S8. Time-course progression of circumferential strains from end-diastole (ED; t = 0 ms) to end-systole (ES; t = 120 ms) at the basal, mid, and apical short-axis (SA) slices of the LV. Regional strains are presented as the average of the strain distribution in each segment of the AHA segmentation plot. Strains are shown for super-resolution reconstruction (SRR) of the LV using two configurations: (A–F) SA images combined with orthogonally sampled long-axis (LA) images (SR-O), and (G–L) SA images combined with radially sampled LA images (SR-R). (A–C, G–I) Strain calculations are also presented for LV volumes reconstructed using the corresponding low-resolution image stacks.

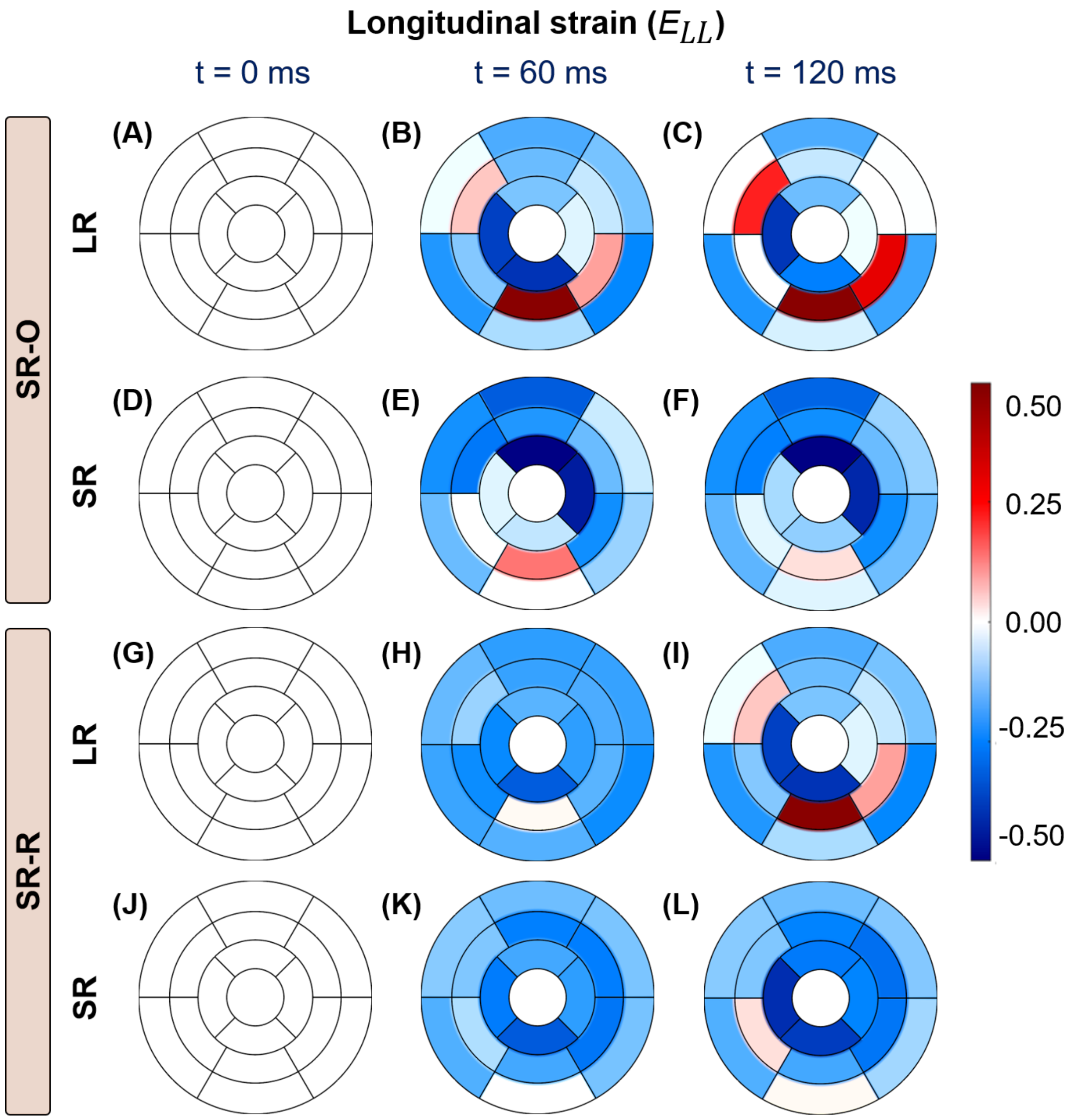

Supplementary Figure S9. Time-course progression of longitudinal strains from end-diastole (ED; t = 0 ms) to end-systole (ES; t = 120 ms) at the basal, mid, and apical short-axis (SA) slices of the LV. Regional strains are presented as the average of the strain distribution in each segment of the AHA segmentation plot. Strains are shown for super-resolution reconstruction (SRR) of the LV using two configurations: (A–F) SA images combined with orthogonally sampled long-axis (LA) images (SR-O), and (G–L) SA images combined with radially sampled LA images (SR-R). (A–C, G–I) Strain calculations are also presented for LV volumes reconstructed using the corresponding low-resolution image stacks.
